# Harnessing Large‐Language Models for Efficient Data Extraction in Systematic Reviews: The Role of Prompt Engineering

**DOI:** 10.1002/cesm.70058

**Published:** 2025-10-27

**Authors:** Molly Murton, Ellie Boulton, Shona Cross, Ambar Khan, Swati Kumar, Giuseppina Magri, Charlotte Marris, David Slater, Emma Worthington, Elizabeth Lunn

**Affiliations:** ^1^ Costello Medical Consulting Limited Cambridge UK; ^2^ Costello Medical Consulting Limited Manchester UK; ^3^ Costello Medical Consulting Limited London UK

**Keywords:** artificial intelligence, data extraction, large‐language model, prompt engineering, systematic literature review

## Abstract

**Introduction:**

Systematic literature reviews (SLRs) of randomized clinical trials (RCTs) underpin evidence‐based medicine but can be limited by the intensive resource demands of data extraction. Recent advances in accessible large‐language models (LLMs) hold promise for automating this step, however testing is limited across different outcomes and disease areas.

**Methods:**

This study developed prompt engineering strategies for GPT‐4o to extract data from RCTs across three disease areas: non‐small cell lung cancer, endometrial cancer and hypertrophic cardiomyopathy. Prompts were iteratively refined during the development phase, then tested on unseen data. Performance was evaluated via comparison to human extraction of the same data, using F1 scores, precision, recall and percentage accuracy.

**Results:**

The LLM was highly effective for extracting study and baseline characteristics, often equaling human performance, with test F1 scores exceeding 0.85. Complex efficacy and adverse event data proved more challenging, with test F1 scores ranging from 0.22 to 0.50. Transferability of prompts across disease areas was promising but varied, highlighting the need for disease‐specific refinement.

**Conclusion:**

Our findings demonstrate the potential of LLMs, guided by rigorous prompt engineering, to augment the SLR process. However, human oversight remains essential, particularly for complex and nuanced data. As these technologies evolve, continued validation of AI tools will be necessary to ensure accuracy and reliability, and safeguarding of the quality of evidence synthesis.

## 1 Introduction

Systematic literature reviews (SLRs) of randomized clinical trials (RCTs) are a cornerstone of evidence‐based medicine [1, 2]. By identifying and synthesizing data across multiple studies, SLRs ensure that healthcare decisions are informed by comprehensive and robust evidence [3, 4]. However, the rapidly growing volume of published literature (in biomedicine and life science, currently over 1 million articles are published each year) [5], not only means that SLRs are increasingly time‐ and resource‐intensive [6, 7], but also that they can rapidly become outdated [8, 9]. The process involves a series of structured steps, beginning with extensive database and gray literature searches. This is followed by dual‐record screening, data extraction and quality assessment, culminating in data synthesis and critical analysis.

Data extraction is often a particularly complex and time‐intensive stage, especially for SLRs within large, evidence‐rich disease areas. It involves meticulous tabulation of detailed information from included studies, including bibliographic details; study and patient characteristics; outcomes (including for different subgroups and timepoints) and quality assessments [10]. Gold standard methodology requires extractions to be performed in duplicate, or at minimum independently verified for accuracy and completeness [4].

Recent advances in artificial intelligence (AI), particularly large‐language models (LLMs), have significant potential to enhance the efficiency of data extraction. LLMs are advanced AI systems, trained on vast amounts of data to understand and generate human‐like speech [11, 12]. They are able to quickly process large volumes of textual data, making them ideal for data extraction tasks. Moreover, the accessibility of LLMs has increased dramatically in recent years, enabling a broader range of users without specialized expertise to leverage them [12]. LLMs are guided by specific inputs, known as “prompts.” “Prompt engineering” involves the deliberate design of prompts, such as specific questions or instructions, to maximize the accuracy, relevance, and usefulness of LLM‐generated responses [13].

However, the performance of LLMs in the context of data extraction is variable. For example, in studies that have assessed the ability of GPT‐4 to extract information from abstracts or full texts, performance was good for study details and patient characteristics, but more limited or simply untested in outcome data [14, 15]. GPT‐3.5 was limited in the extraction of data from full text articles, especially data presented in figures [16]. Meanwhile, another LLM, Claude 2, displayed high accuracy in the extraction of primary outcome data in a single disease area (although data of interest was almost always included in the studies, limiting the chance of confabulations) [17]. Despite some promising results, the evidence base is largely limited to small, domain‐specific studies, or those extracting a limited number of data elements. Notably, there is a lack of studies assessing extractions of large numbers of data points, along with lack of testing for generalizability of results across different disease areas/domains. Furthermore, concerns regarding the risks of inaccuracies and confabulations remain significant, as any misrepresentations would critically undermine the crucial requirement for accuracy and completeness of information in evidence synthesis.

To address these challenges and harness the benefits of LLM‐assisted data extraction requires clear frameworks for AI usage; clarity on human‐in‐the‐loop (HITL) involvement; and investment in prompt engineering. Rigorous testing and validation of AI‐based approaches before widespread implementation is also vital.

This paper presents methods and results for prompt engineering to guide an LLM in extracting a large amount of data from RCTs across diverse disease areas. We evaluate the accuracy and efficiency of AI‐assisted data extraction compared to traditional human extraction. Our research seeks to explore the benefits and limitations of AI in enhancing the extraction process, ultimately aiming to improve reliability and efficiency. Our findings have significant implications for evidence‐based medicine, potentially enabling more efficient decision‐making by addressing some current limitations.

## 2 Methods

### 2.1 Model Selection

Several LLMs were considered for this study. GPT‐4o, accessible through the OpenAI Application Programming Interface (API), was selected due to its widespread adoption and ease of access. It was integrated into a purpose‐built AI assistant within a web application with a manual interface, embedded in an enterprise environment to provide contextual and document‐based support. GPT‐4o has a temperature range of 0–2, with low values producing more deterministic results (i.e., the most probable word is always chosen) and high values producing more creative outputs. For this study, a temperature setting of 0.7 was selected, as a typical default setting that ensures balanced output.

### 2.2 Overview

**Figure 1:**
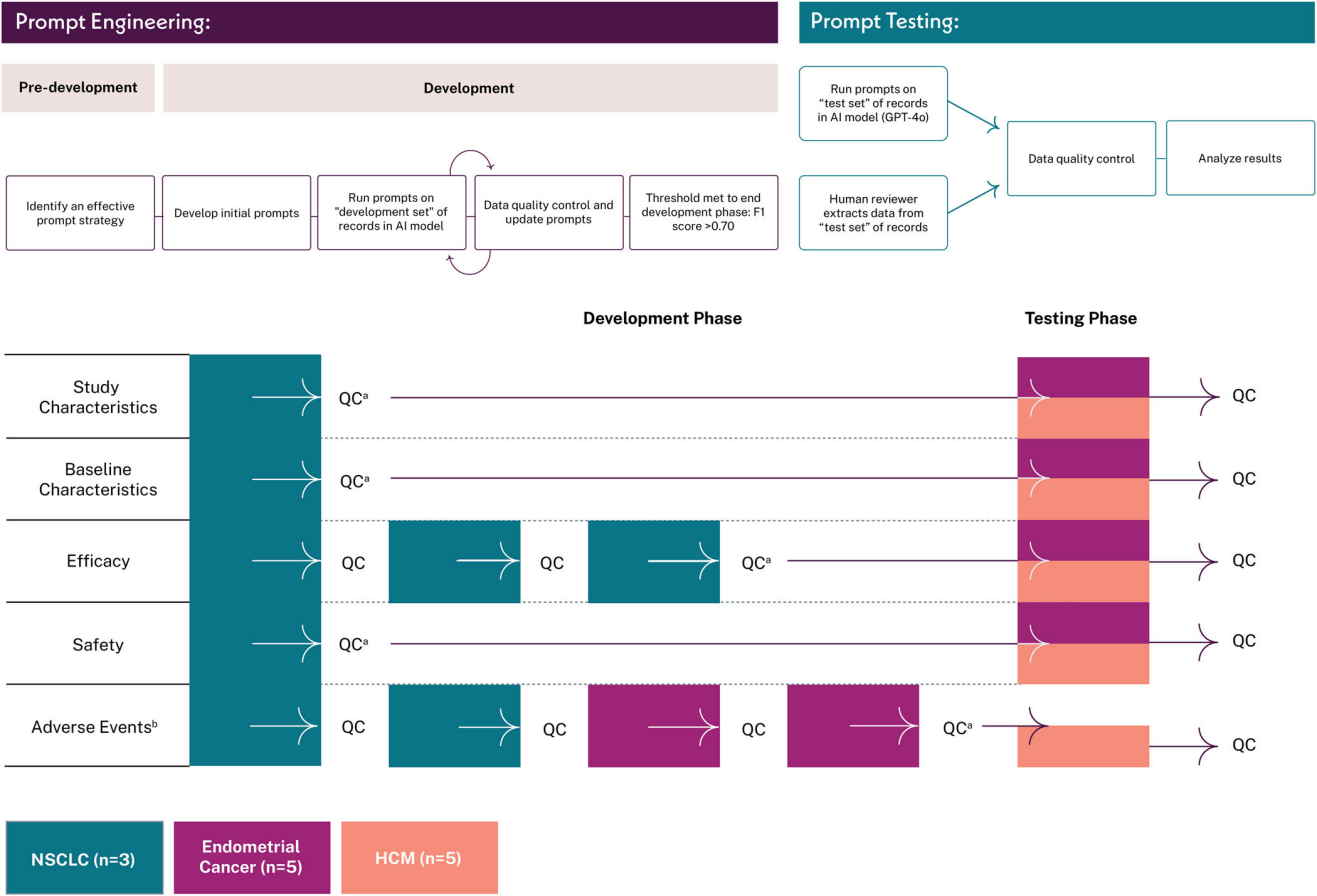
Summary of prompt engineering workflow. Footnote: Each colored box represents a prompt iteration or test. ^a^Represents the point at which the F1 score reached the desired threshold (> 0.70) or further improvements were not achievable. ^b^During the second NSCLC prompt iteration only two NSCLC studies were used. Abbreviations: AI, artificial intelligence; HCM, hypertrophic cardiomyopathy; NSCLC, non‐small cell lung cancer; QC, quality control.

The approach to prompt engineering is summarized in Figure 1. Briefly, there were three stages: (1) Predevelopment, (2) Development and (3) Testing. Three disease areas were selected: non‐small cell lung cancer (NSCLC), endometrial cancer and hypertrophic cardiomyopathy (HCM). For each disease area, a pre‐defined extraction grid was created to specify the information to be captured. Each grid was divided into sections for study characteristics, baseline characteristics, efficacy outcomes, safety outcomes, and adverse events (AEs). Each grid contained > 500 individual data fields (> 1500 data fields in total across the three disease areas). A convenience‐based sample of articles was chosen from each disease area, including coverage of conference abstracts, full‐text publications (with and without supplementary files), and clinical trial records. No data preprocessing was carried out on the articles; the PDFs were uploaded alongside each prompt.

#### 2.2.1 Predevelopment Phase

The predevelopment phase aimed to identify the most effective prompting strategy for efficient and accurate data extraction. Five articles reporting on RCTs in NSCLC served as the starting dataset. Initially, separate prompts were created for each individual data field (i.e., each prompt was designed to extract a single data point; an example of this prompt structure is provided in Supporting Information S1: Appendix 2), and each run in a new session via API. However, this approach had limited success as it was computationally inefficient, provided minimal context to the model and could not handle between‐study variability such as differing numbers of treatment arms or patient subgroups. Therefore, this approach was discontinued in favor of developing larger, composite prompts and prompt chaining. The large prompts instructed the model to populate multiple cells of the extraction table simultaneously, broken down by data category or subcategory. This method was substantially more effective and was taken forward into the development and testing phases.

#### 2.2.2 Development Phase

The purpose of the development phase was to iteratively refine a set of prompts through repeated testing and modification until a performance threshold was met or no further improvements were observed. Due to resource constraints, three of the five NSCLC articles used in the predevelopment phase were randomly selected for use in the development phase. Prompt structures were flexible and varied based on the section of the extraction grid. However, common elements included initial context setting, use of clear language and inclusion of relevant examples.

After each iteration, the AI‐generated output was compared against a human extraction of the same study by a human reviewer. The human extraction was conducted by one reviewer and independently verified by a second human reviewer, with discrepancies adjudicated by a third individual if required. The AI output was marked according to the following criteria:

- **Correct (true positive)**: Accurate data (minor formatting issues allowed).
- **Incorrect (false positive)**: Incorrect or misinterpreted data.
- **Missing (false negative)**: Failure to identify reported data (e.g., extracting “not reported” when the data point was available).

F1 scores were then calculated as:

$$F1=\;\frac{2\times \text{precision}\times \text{recall}}{\text{precision}+\text{recall}}$$

Footnote: Precision = (true positives)/(true positives + false positives). Recall = (true positives)/(true positives + false negatives). F1 scores can range from 0 to 1.

F1 scores range from 0 to 1, where a higher score signifies a better overall performance in terms of balance between precision and recall [18]. A high F1 score indicates that the prompt accurately identified relevant data (recall) while minimizing incorrect outputs (precision). A pre‐specified target F1 score of > 0.70 was set, as this is commonly considered to represent a good benchmark in practice [18, 19]. If F1 > 0.70 was not achieved, the next iteration was conducted, focusing on refining areas of suboptimal AI performance. AI assistance was sought for prompt optimization where necessary.

For efficacy data, three different F1 scores were calculated corresponding to three levels of data complexity:

- **Simple – primary efficacy**: data for the primary timepoint, outcome assessor, analysis set and population.
- **Moderate – overall efficacy without subgroup data**: all data excluding subgroups.
- **Complex – overall efficacy**: all relevant data, including secondary/tertiary timepoints, outcomes and subgroups.

For AE prompts, the F1 threshold was not met in any iteration, leading to a decision to develop AE prompts in a different disease context. Five endometrial cancer articles were used for AE prompt development.

#### 2.2.3 Testing Phase

The testing phase assessed the generalizability of the final developed prompts to new, unseen data in the other two disease areas selected for this study. Final prompts were tested on five studies reporting on RCTs in HCM and five studies reporting on RCTs in endometrial cancer, with only minimal modification to incorporate disease‐specific information. F1 scores for AI‐extracted data were then compared against scores for human extraction of the same studies. Precision and recall were also calculated. The final prompts are presented in Supporting Information S1: Appendix 2.

## 3 Results

### 3.1 Development Phase

**Figure 2:**
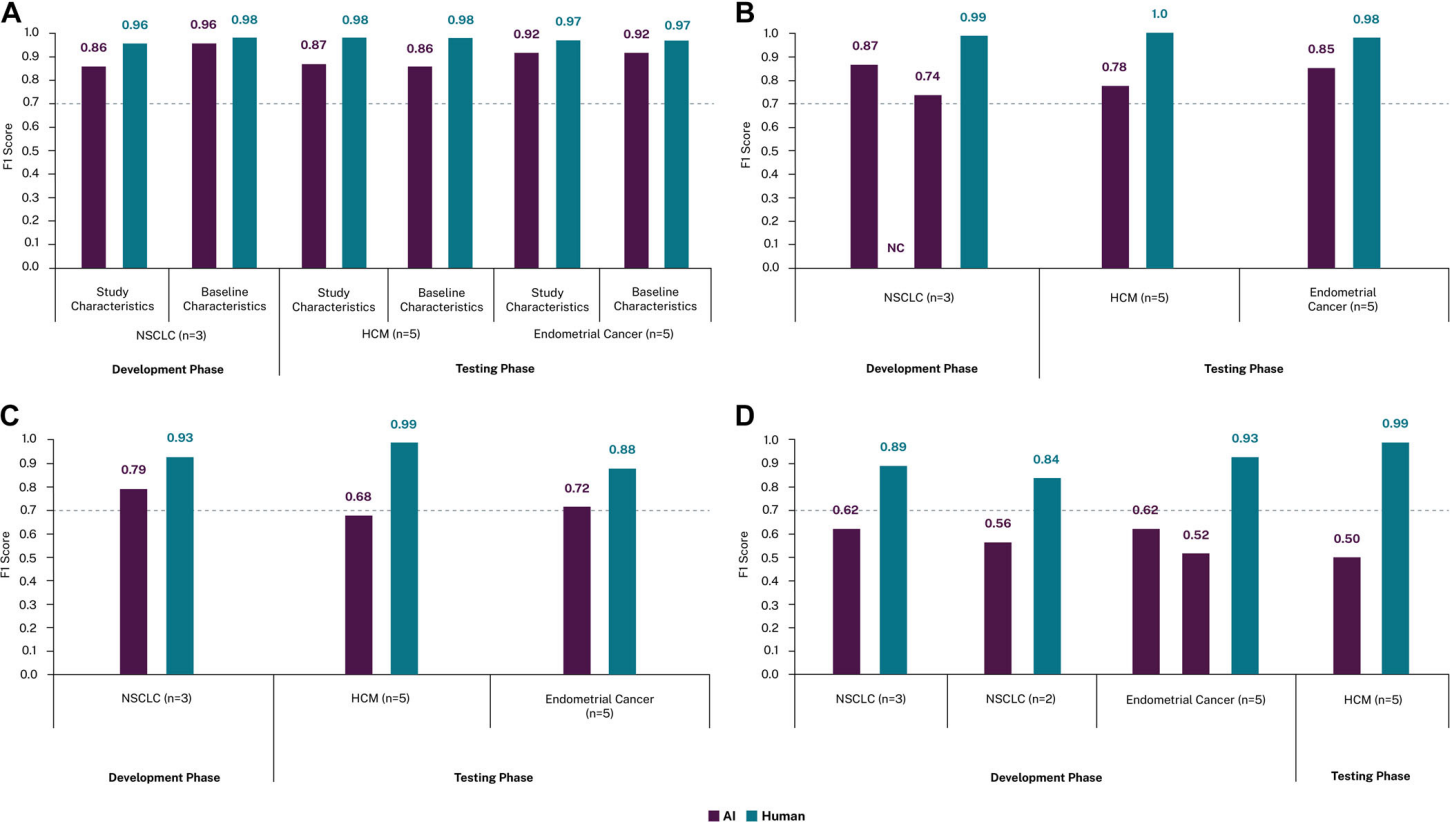
Prompt development and testing F1 scores for each extraction grid section in each disease area: (A) Baseline and Study Characteristics; (B) Efficacy (primary data only); (C) Safety and (D) Adverse Events. Footnote: NC refers to “not calculated” as the model failed to consistently retrieve subgroup data (identical prompts applied to the same publications yielded varying results with differing levels of subgroup data extracted), and therefore, an F1 score was not calculated for this iteration. Abbreviations: AI, artificial intelligence; BLC, baseline characteristics; HCM, hypertrophic cardiomyopathy; NC, not calculated; NSCLC, non‐small cell lung cancer.

Across all papers and extraction fields, > 25,000 data elements were extracted in the development phase. F1 scores for each prompt iteration during development are detailed in Table 1 and Figure 2. Precision and recall are presented in Supporting Information S1: Appendix 1, Tables A1 and A2.

**Table 1:** Development phase results: F1 scores^a^.

	*N* data elements (fields)	F1 scores				
		Human	Iteration 1	Iteration 2	Iteration 3	Iteration 4
Study characteristics	87 (42)	0.96	0.86	—	—	—
Baseline characteristics	1077 (170)	0.98	0.96	—	—	—
Primary efficacy	1449 (149)	0.99	0.87	Not calculated^c^	0.74	—
Overall efficacy without subgroup	6572 (149)	0.99	0.69		0.33	—
Overall efficacy	9587 (149)	0.99	Not calculated^b^		0.28	—
Safety	583 (58)	0.93	0.79	—	—	—
AEs: NSCLC development	2272 (109)	Iteration 1: 0.89	0.62	0.56^d^	—	—
		Iteration 2: 0.84^d^				
AEs: endometrial cancer development (*n* = 5)	5716 (196)	0.93	—	—	General grade:^e^ 0.62	Specific grade:^f^ 0.52

Abbreviations: AE, adverse event; NSCLC, non‐small cell lung cancer.

^a^ Three NSCLC studies were used unless otherwise stated.

^b^ The prompt did not include a column for subgroup data in this iteration.

^c^ The model failed to consistently retrieve subgroup data (identical prompts applied to the same publications yielded varying results with differing levels of subgroup data extracted), and therefore, an F1 score was not calculated for this iteration.

^d^ Only two NSCLC studies were used in this iteration.

^e^ A broad prompt was employed to encourage the model to extract all adverse events of any reported grade. An example of how these AEs might be reported was provided (e.g., “Grade 3,” “Grade 3–4,” or “any grade”).

^f^ Separate, specific prompts were utilized to encourage the model to extract reported AEs corresponding to each individual grade (e.g., Grade 1), if present in the article.

For study characteristics, baseline characteristics and safety outcomes, only one iteration of prompts was required. The F1 scores were 0.86, 0.96, and 0.79, respectively. The study characteristics prompt was straightforward, with all details requested in one comprehensive query. Baseline characteristics and safety sections both required several smaller sub‐prompts to capture all aspects of the data. An excerpt of the prompt for study characteristics is presented below:

“Extract the results from this publication into a single table with the following headings: Study name (the first author surname and the year the paper was published e.g., Smith 2023, if the clinical trial has a name i.e., Study 123 report this instead); Single‐center/multi‐center (State whether the study was 'single‐center' or 'multi‐center'. State the term only); Study phase (State the study phase, out of: Phase I, Phase II, Phase III, Phase IV. State one answer); …”

For efficacy, three iterations of prompts were required. Extracting primary efficacy data consistently yielded high performance, with F1 scores exceeding 0.70 in all iterations (ranging from 0.74 to 0.87). However, when attempting to include more complex and comprehensive data, such as additional timepoints, analysis sets or subgroups, performance deteriorated. The first iteration that attempted to incorporate subgroup data was discarded without F1 score calculation, as recall was too low (i.e., only a very limited amount of subgroup data were retrieved). Recall was improved by chaining smaller sub‐prompts that focused on explicit retrieval of subgroups and additional data, for example:

“Now make table rows, using the same headings and format for different timepoints. A table is not needed if there are no additional data.”

“Now make table rows, using the same headings and format for different assessors so that 'Investigator' or 'Independent/central review' are extracted if needed. A table is not needed if there are no additional data.”

“Now make table rows, using the same headings for different analysis sets. A table is not needed if there are no additional data.”

“Now identify all possible subgroups reported across the entirety of the publications, including supplementary tables and figures, to ensure comprehensive representation. Examples of potential subgroups include: sex (male or female), age, ECOG performance status, smoking status, severity of disease, tumor stage, mutation status, lines of therapy, race. Make rows, using the same headings for all identified subgroups. A table is not needed if there is no additional data.”

However, improved recall was at the expense of lower accuracy and increased confabulations. This included extraction of data, such as timepoints and subgroups, that were not present in the source. It also increased the time taken to run the prompts. This resulted in a low F1 score for overall efficacy (0.28). Therefore, the decision to progress to the testing phase was based on performance for primary efficacy data only.

For AEs, four prompt iterations were performed, two iterations across NSCLC (*n* = 3 studies for iteration 1; *n* = 2 studies for iteration 2) and two iterations across endometrial cancer (*n* = 5 studies). The model could not accurately extract different AE grades, particularly when they were grouped (e.g., Grade 1–2). Prompts that explicitly requested specific AE grades (e.g., Grade 1 vs. Grade 2 vs. Grade 3) yielded F1 scores of 0.62 and 0.56, while non‐explicit prompts (that left the model to define the grade) achieved similar performance (0.62). Ultimately, it was not possible to achieve an F1 score > 0.70 for AE data. The non‐explicit prompts were taken forward for testing, as they did not require prior knowledge of the different grades reported in studies. The prompt took the following structure:

“AE Grade (First check for a table reporting any AEs which is likely to report the AE grades. Include all AE grades reported: these could be individual grades or categorized for example, any or all grades, grade X, GX, Grade X‐X, Grade X/X, Grade ≥ X [where X is any number between 0 and 5], serious, moderate, any grade. These are only a few examples so present this data how the publication reports it).”

As for baseline characteristics, safety and efficacy outcomes prompts were divided into smaller sub‐prompts, each retrieving information on several specific AEs (e.g., one prompt queried for information on ~10 specific AEs, such as abdominal pain, adrenal insufficiency, alopecia, anemia, anorexia, arthralgia, colitis).

### 3.2 Testing Phase

Across all papers and extraction fields, > 40,000 data elements were extracted in the testing phase (F1 scores are presented in Table 2 and Figure 2 for both AI and human extractions; precision and recall are presented in Supporting Information S1: Appendix 1, Tables A3 and A4). Overall, AI performance was variable, being comparable or superior to human extraction in some categories, but inferior in others.

**Table 2:** Testing phase results: F1 scores.

	Endometrial Cancer (*N* = 5)				HCM (*N* = 5)			
	*N* data elements (fields)	F1 scores			*N* data elements (fields)	F1 scores		
		AI	Human	Difference		AI	Human	Difference
Study characteristics	180 (67)	0.92	0.97	−0.05	156 (50)	0.87	0.98	−0.11
Baseline characteristics	2130 (213)	0.95	0.82	+0.13	2106 (152)	0.86	0.99	−0.13
Primary efficacy	1342 (153)	0.85	0.98	−0.13	2292 (152)	0.78	1.0	−0.22
Overall efficacy without subgroup	4326 (153)	0.39	0.90	−0.51	6727 (152)	0.37	1.0	−0.63
Efficacy (overall)	8800 (153)	0.30	0.92	−0.62	12,206 (152)	0.22	1.0	−0.78
Safety Data	1330 (97)	0.72	0.88	−0.16	704 (68)	0.68	0.99	−0.31
AE Data	NA^a^	NA^a^	NA^a^	NA^a^	2772 (133)	0.50	1.0	−0.50

*Note:* 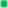 difference > 0 (i.e., AI score was better than the human score); 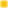 difference between −0.5 and 0 (i.e., AI score was worse than the human score; 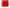 difference ≤ −0.5 (i.e., AI score was considerably worse than the human score).

Abbreviations: AI, artificial intelligence; AE, adverse event; HCM, hypertrophic cardiomyopathy; NA, not applicable.

^a^ The AE prompts were still in development phase when using the endometrial cancer studies and so no test F1 score is available.

Test AI extraction performance was strong for study characteristics and baseline characteristics across both unseen datasets (endometrial cancer and HCM). F1 scores for study characteristics were 0.92 for endometrial cancer and 0.87 for HCM; F1 scores for baseline characteristics were 0.95 for endometrial cancer and 0.86 for HCM. Notably, AI outperformed human extraction for baseline data in endometrial cancer (F1 0.95 vs. 0.82), whereas AI was similar to human extractions for study characteristics (F1 0.92 vs. 0.97).

**Figure 3:**
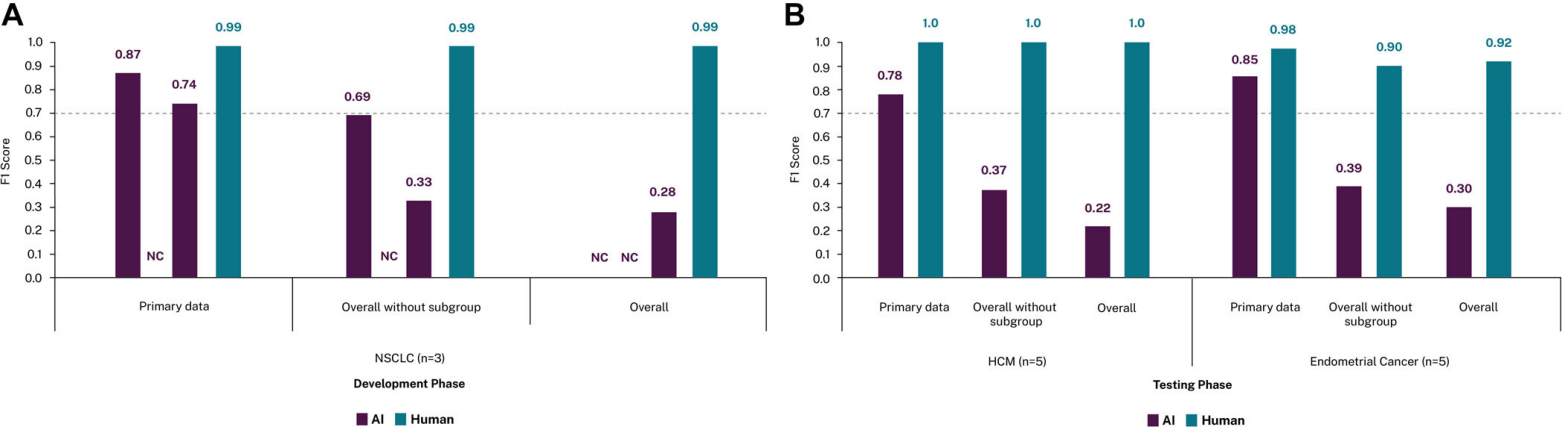
(A) Prompt development and (B) testing F1 scores for efficacy data, by complexity. Footnote: NC refers to “not calculated.” For the first iteration in the development phase the overall data F1 score was NC as the prompt did not include a column for subgroup data. The second iteration development phase F1 scores were not calculated as the model failed to consistently retrieve subgroup data with identical prompts applied to the same publications yielding varying results with differing levels of subgroup data extracted. Abbreviations: AI, artificial intelligence; HCM, hypertrophic cardiomyopathy; NC, not calculated; NSCLC, non‐small cell lung cancer.

Performance on efficacy data varied with complexity. The LLM achieved high F1 scores of 0.85 in endometrial cancer and 0.78 in HCM for primary efficacy data. However, scores were substantially lower for overall efficacy data (F1 0.30 and 0.22, respectively), mirroring the development phase results. Figure 3 summarizes the F1 scores for primary efficacy data from the development and testing phases. Meanwhile, performance on safety data was moderate (F1 0.72 for endometrial cancer, 0.68 for HCM). However, for AEs, an F1 of 0.50 was achieved for HCM, reflecting persistent challenges in accurate extraction of detailed AE data.

Across all outcomes, AI performed better on endometrial cancer studies compared with HCM studies. By contrast, the reverse was true for human extractions, where F1 scores were higher in HCM. The greatest increase in F1 score from the final development prompt to the testing phase was observed for primary efficacy data in endometrial cancer (+0.11), while the largest decreases were in HCM for baseline characteristics (−0.10), safety (−0.11) and AEs (−0.12). An overview of the F1 scores from the development and testing phases for study characteristics, baseline characteristics, efficacy, safety and adverse events outcomes are summarized in Figure 2, while the difference in F1 scores from last development iteration to testing phase are presented in Table 3. Corresponding precision and recall results are presented in Supporting Information S1: Appendix 1, Tables A5 and A6.

**Table 3:** Difference in F1 scores from last development iteration to testing phase.

	Final development set^a^	Test set endometrial cancer (*N* = 5)	Difference versus development set	Test set HCM (*N* = 5)	Difference versus development set
Study characteristics	0.86	0.92	+0.06	0.87	+0.01
Baseline characteristics	0.96	0.95	−0.01	0.86	−0.10
Primary efficacy	0.74	0.85	+0.11	0.78	+0.04
Overall efficacy without subgroup	0.33	0.39	+0.06	0.37	+0.04
Overall efficacy	0.28	0.30	+0.02	0.22	−0.06
Safety	0.79	0.72	−0.07	0.68	−0.11
AEs	0.62	NA^b^	NA^b^	0.5	−0.12

*Note:* 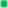 difference > 0 (i.e., test score was better than the development score); 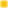 difference between −0.5 and 0 (i.e., test score was worse than the development score; 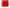 difference ≤−0.5 (i.e., test score was considerably worse than the development score).

Abbreviations: AI, artificial intelligence; AE, adverse event; HCM, hypertrophic cardiomyopathy; NA, not applicable.

^a^ Three NSCLC studies were used in the last development phase for all outcomes except for AE data which used five endometrial studies.

^b^ The AE prompts were still in development phase when using the endometrial cancer studies and so no test F1 score is available.

**Figure 4:**
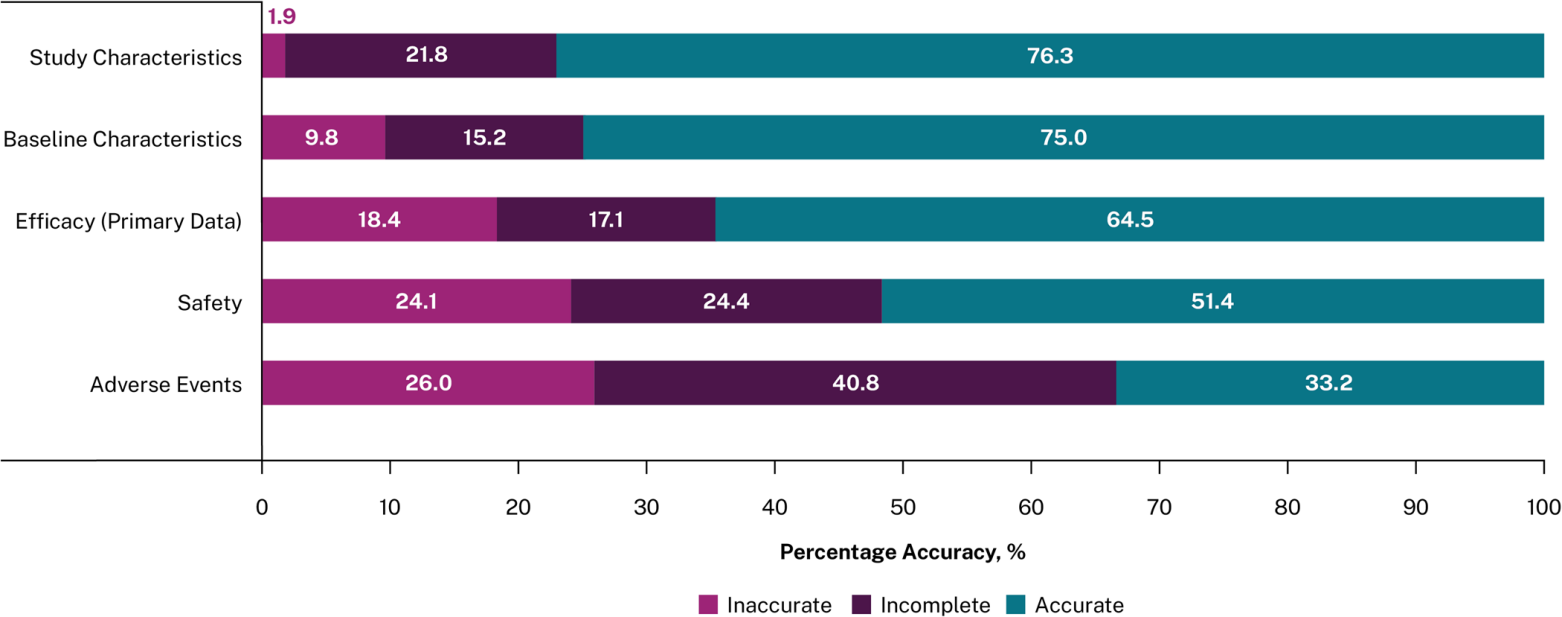
% accuracy for HCM tests. Footnote: Five articles from the HCM test set was used for this analysis. “Incomplete”: AI model failed to identify data point that was present in the article; “Inaccurate”: AI model extracted data point incorrectly; “Accurate”: AI model correctly identified data point. Accuracy was assessed separately for different sections of the extraction grid. Totals may not sum to 100% due to rounding. Abbreviations: AI, artificial intelligence; HCM, hypertrophic cardiomyopathy.

Finally, Figure 4 provides a breakdown of the extraction accuracy for each outcome in the HCM test set. The proportion of accurate data was highest for study characteristics (76.3%) followed by baseline characteristics (75.0%). It was lowest for safety (51.4%) and AEs (33.2%). This reflects the F1 scores seen across the different outcomes.

## 4 Discussion

To our knowledge, this is the first study to optimize data extraction prompts for one disease area and then test their performance on unseen RCT data across multiple disease areas.

Results for the unseen data were varied, ranging from excellent to poor. In one instance, AI‐assisted extractions surpassed human performance (baseline characteristics, endometrial cancer). Additionally, F1 scores for study characteristics and primary efficacy data were comparable between AI and humans. These results clearly demonstrate AI's ability to accurately extract unseen data using prompts developed for an unrelated disease. On the other hand, performance was less encouraging for safety data, AEs and complex subgroup efficacy data. This is consistent with other studies that report better results for simple data and limitations with more complex outcomes [14, 17].

The success in extracting study and baseline characteristics may be attributed to their relative simplicity, often involving a limited number of data points. Conversely, outcome data (efficacy, safety and AEs) are inherently more complex, often including multiple timepoints, definitions and subgroups. This greatly increases the number of data elements and thus difficulty of the extraction. Relatedly, our findings suggest that one uniform prompt structure is not suitable for all outcome types. For example, while a single composite prompt worked well for study characteristics, other sections required multiple smaller sub‐prompts. For complex efficacy data, sub‐prompts effectively increased recall but also led to confabulations, lower precision and longer processing time, highlighting the key trade‐offs. These findings clearly indicate that AI cannot replace human involvement in data extractions; human oversight is paramount to ensure data integrity and reliability. In particular, the need to avoid confabulations cannot be understated, as they may lead to the propagation of serious misinformation, undermine trust in data and ultimately lead to misguided decision‐making. Some of the poor performance may stem from innate model capabilities. Yet as AI becomes more widely adopted in professional settings, advancements in LLMs are expected, and may soon enable effective extraction of more complex datasets [20].

During testing, AI performed moderately more accurately on endometrial cancer studies than on HCM. This may be because prompts were initially developed using NSCLC studies, as oncology studies often report similar outcomes. Notably, however, human extraction performance also varied by disease area, and was better for HCM than endometrial cancer. This is indicative of how factors specific to the disease area may extrinsically influence extraction accuracy, and that caution should be taken when comparing F1 scores across disease areas. As such, applying prompts across different disease areas requires careful refinement, as a direct transfer may not maintain performance. However, given the significant time investment required for prompt engineering, such effort should be justified by meaningful improvements in performance. Ultimately, AI‐assisted extraction only offers efficiency gains for the SLR process if it generates faster and more reliable outcomes than traditional human extractions. This underscores the importance of designing prompts that are both effective and computationally efficient.

## 5 Strengths and Limitations

A key strength of this study is the use of an accessible LLM, GPT‐4o, which is readily available for use by anyone, without the need for specialist knowledge. Furthermore, the extraction of a large number of data elements reflects the complexity often encountered in real‐world SLRs of RCTs. Additionally, conducting prompt development in one disease area and testing across two distinct unseen disease areas increases the generalizability of our findings.

However, there were some limitations to our work. While a large number of data elements were included, the study sample size was relatively small, limited to a maximum of five studies per disease area. This may not capture the extent of between‐study variability in large SLRs. Additionally, only a single model was tested; exploring different models and tuning hyperparameters could provide further insights and optimize performance. The temperature setting of 0.7 was chosen as a default value, however, this is not optimal for factual tasks like data extraction, as values above 0 encourage more variation in responses. Future work should ensure that a temperature setting of 0 is used, to minimize randomness in responses. Additionally, as no preprocessing steps were applied to PDFs, document quality and formatting may have impacted AI extraction performance. While the AI‐generated output was compared with a human extraction completed by two human reviewers, thereby reducing the risk of error compared with checking against the PDF, the comparison was only performed by one reviewer which may affect the reliability of results. Performance evaluation primarily focused on accuracy metrics, and more comprehensive assessments of efficiency and workflow impact are required. Lastly, our methodology involved manual steps including uploading PDFs, repeated prompting, and copy/pasting of results, which may limit the scalability of the process.

## 6 Recommendations

Future research should include larger, more diverse datasets to better understand AI performance across different contexts. More sophisticated elements could be integrated into prompting approaches, such as utilizing specifically fine‐tuned LLMs, controlling output formats (e.g., JSON), or developing fully automated SLR workflows that embed AI extraction directly into existing systems.

Based on our findings, we propose several recommendations for effective and responsible use of LLMs for data extraction in SLRs:

1. AI cannot fully replace human extraction–HITL approaches are essential for maintaining the quality and integrity of evidence synthesis.
2. Prompt engineering should be a continuous process, refined iteratively to balance recall, precision, and efficiency. As AI models evolve, developing more sophisticated prompts tailored to specific data types and contexts will be essential.
3. Integration of AI into the SLR workflow should focus on automation and specialized tools to minimize additional review time, enabling faster and more reliable data extraction at scale.
4. Broader validation across multiple reviews and independent datasets will be necessary to confirm the reproducibility and robustness of these methods, ultimately facilitating greater adoption of AI in evidence synthesis.

## 7 Conclusion

This study highlights both the potential and current limitations of AI in the data extraction of RCTs. While AI can match human extraction in certain areas, its effectiveness varies by data complexity and human oversight remains vital. Furthermore, while transferability of prompts across disease areas is promising, tailored prompt strategies should be considered for best performance, if the time investment is justified. Future efforts should focus on larger datasets and continued testing in more advanced AI models, all aimed at fully harnessing AI's promise to optimize SLR workflows.

## Author Contributions

**Molly Murton:** conceptualization, investigation, writing – original draft, methodology, writing – review and editing, supervision, formal analysis. **Ellie Boulton:** project administration, investigation, writing – original draft, writing – review and editing, formal analysis, methodology. **Shona Cross:** writing – review and editing, methodology, investigation, formal analysis. **Ambar Khan:** investigation, writing – review and editing, methodology, formal analysis. **Swati Kumar:** investigation, methodology, formal analysis, writing – review and editing. **Giuseppina Magri:** investigation, writing – original draft, writing – review and editing, methodology, formal analysis. **Charlotte Marris:** investigation, writing – review and editing, methodology, formal analysis. **David Slater:** writing – original draft, writing – review and editing, investigation, methodology, formal analysis. **Emma Worthington:** investigation, methodology, formal analysis, writing – review and editing. **Elizabeth Lunn:** conceptualization, investigation, writing – original draft, writing – review and editing, supervision, methodology, formal analysis.

## Disclosure

The authors are employees of Costello Medical Consulting Limited.

## Peer Review

The peer review history for this article is available at https://www.webofscience.com/api/gateway/wos/peer-review/10.1002/cesm.70058.

## Supporting information

Prompt Engineering for LLM Data Extraction Appendix 01Oct25.

## Data Availability

The data that support the findings of this study are available from the corresponding author upon reasonable request.

## References

[1] M. H. Murad , N. Asi , M. Alsawas , and F. Alahdab , “New Evidence Pyramid,” Evidence Based Medicine 21 (2016): 125–127. 27339128 10.1136/ebmed-2016-110401 PMC4975798

[2] J. McSharry “What Health Evidence Can We Trust When We Need It Most?”. Evidently Cochrane blog. 19 January 2023. https://www.cochrane.org/news/what-health-evidence-can-we-trust-when-we-need-it-most. Accessed: 22/05/25.

[3] J. Chandler , M. Cumpston , T. Li , et al., Cochrane Handbook for Systematic Reviews of Interventions (Hoboken: Wiley, 2019).

[4] Centre for Reviews and Dissemination ., Systematic Reviews: CRD's Guidance for Undertaking Reviews in Health Care (York: Centre for Reviews and Dissemination, University of York, 2008).

[5] R. González‐Márquez , L. Schmidt , B. M. Schmidt , P. Berens , and D. Kobak , “The Landscape of Biomedical Research,” Patterns 5 (2024): 100968. 39005482 10.1016/j.patter.2024.100968 PMC11240179

[6] B. Smela , M. Toumi , K. Świerk , K. Gawlik , E. Clay , and L. Boyer , “Systematic Literature Reviews Over the Years,” Journal of Market Access & Health Policy 11 (2023): 2244305. 37614556 10.1080/20016689.2023.2244305 PMC10443963

[7] R. Borah , A. W. Brown , P. L. Capers , and K. A. Kaiser , “Analysis of the Time and Workers Needed to Conduct Systematic Reviews of Medical Interventions Using Data From the PROSPERO Registry,” BMJ Open 7 (2017): e012545. 10.1136/bmjopen-2016-012545 PMC5337708 28242767

[8] K. G. Shojania , M. Sampson , M. T. Ansari , J. Ji , S. Doucette , and D. Moher , “How Quickly Do Systematic Reviews Go Out of Date? A Survival Analysis,” Annals of Internal Medicine 147 (2007): 224–233. 17638714 10.7326/0003-4819-147-4-200708210-00179

[9] M. Z. Andersen , S. Gülen , S. Fonnes , K. Andresen , and J. Rosenberg , “Half of Cochrane Reviews Were Published More Than 2 Years After the Protocol,” Journal of Clinical Epidemiology 124 (2020): 85–93. 32413390 10.1016/j.jclinepi.2020.05.011

[10] B. Nussbaumer‐Streit , M. Ellen , I. Klerings , et al., “Resource Use During Systematic Review Production Varies Widely: A Scoping Review,” Journal of Clinical Epidemiology 139 (2021): 287–296. 34091021 10.1016/j.jclinepi.2021.05.019

[11] M. Raza , Z. Jahangir , M. B. Riaz , M. J. Saeed , and M. A. Sattar , “Industrial Applications of Large Language Models,” Scientific Reports 15 (2025): 13755. 40258923 10.1038/s41598-025-98483-1 PMC12012124

[12] F. Chiarello , V. Giordano , I. Spada , S. Barandoni , and G. Fantoni , “Future Applications of Generative Large Language Models: A Data‐Driven Case Study on ChatGPT,” Technovation 133 (2024): 103002.

[13] L. Wang , X. Chen , X. Deng , et al., “Prompt Engineering in Consistency and Reliability With the Evidence‐Based Guideline for LLMs,” NPJ Digital Medicine 7 (2024): 41. 38378899 10.1038/s41746-024-01029-4 PMC10879172

[14] Y. Li , S. Datta , M. Rastegar‐Mojarad , et al., “Enhancing Systematic Literature Reviews With Generative Artificial Intelligence: Development, Applications, and Performance Evaluation,” Journal of the American Medical Informatics Association 32 (2025): 616–625. 40036547 10.1093/jamia/ocaf030 PMC12005633

[15] Q. Khraisha , S. Put , J. Kappenberg , A. Warraitch , and K. Hadfield , “Can Large Language Models Replace Humans in Systematic Reviews? Evaluating GPT‐4's Efficacy in Screening and Extracting Data From Peer‐Reviewed and Grey Literature in Multiple Languages,” Research Synthesis Methods 15 (2024): 616–626. 38484744 10.1002/jrsm.1715

[16] A. Alshami , M. Elsayed , E. Ali , A. E. E. Eltoukhy , and T. Zayed , “Harnessing the Power of ChatGPT for Automating Systematic Review Process: Methodology, Case Study, Limitations, and Future Directions,” Systems 11 (2023): 351.

[17] G. Gartlehner , L. Kahwati , R. Hilscher , et al., “Data Extraction for Evidence Synthesis Using a Large Language Model: A Proof‐of‐Concept Study,” Research Synthesis Methods 15 (2024): 576–589. 38432227 10.1002/jrsm.1710

[18] N. Van Otten F1 Score The Ultimate Guide: Formulas, Explanations, Examples, Advantages, Disadvantages, Alternatives & Python Code, 2023.

[19] M. Senter Understanding and Calculating the F1 Score in ML.

[20] N. Gillespie , S. Lockey , and T. Ward Trust, Attitudes and Use of Artificial Intelligence. 2025.

