# Supplementary material for: Harnessing Large‐Language Models for Efficient Data Extraction in Systematic Reviews: The Role of Prompt Engineering
Source: Cochrane Evid Synth Methods. 2025 Oct 27;3(6):e70058. doi: 10.1002/cesm.70058 (PMC12559671; doi:10.1002/cesm.70058)
Supplement: Supplementary file 1 — Prompt Engineering for LLM Data Extraction Appendix 01Oct25. [file CESM-3-e70058-s001.docx]

APPENDIX 1: Precision and Recall

Table A1. Development phase results: precision^a^

|  | **N data elements (fields)** | **Precision scores** | | | | |
| --- | --- | --- | --- | --- | --- | --- |
|  |  | **Human** | **Iteration 1** | **Iteration 2** | **Iteration 3** | **Iteration 4** |
| **Study characteristics** | 87 (42) | 0.97 | 0.93 | - | - | - |
| **Baseline characteristics** | 1,077 (170) | 0.99 | 0.98 | - | - | - |
| **Primary efficacy** | 1,449 (149) | 1.00 | 0.93 | Not calculated^c^ | 0.70 | - |
| **Overall efficacy without subgroup** | 6,572 (149) | 0.99 | 0.93 |  | 0.21 | - |
| **Overall efficacy** | 9,587 (149) | 1.0 | Not calculated^b^ |  | 0.19 | - |
| **Safety** | 583 (58) | 0.98 | 0.87 | - | - | - |
| **AEs: NSCLC development** | 2,272 (109) | Iteration 1: 0.99  Iteration 2: 0.99^d^ | 0.97 | 0.56 (0.54, 0.57)^d^ | - | - |
| **AEs: endometrial cancer development (n=5)** | 5,716 (196) | 0.97 | - | - | General grade:^e^ 0.80 | Specific grade:^f^ 0.41 |

**Footnote:** ^a^ Three NSCLC studies were used unless otherwise stated. ^b^ The prompt did not include a column for subgroup data in this iteration. ^C^ The model failed to consistently retrieve subgroup data (identical prompts applied to the same publications yielded varying results with differing levels of subgroup data extracted), and therefore, a precision score was not calculated for this iteration. ^d^ Only two NSCLC studies were used in this iteration. ^e^ A broad prompt was employed to encourage the model to extract all adverse events of any reported grade. An example of how these AEs might be reported was provided (e.g., 'Grade 3', 'Grade 3-4', or 'any grade'). ^f^ Separate, specific prompts were utilized to encourage the model to extract reported AEs corresponding to each individual grade (e.g., Grade 1), if present in the article. **Abbreviations:** AE, adverse event; NSCLC, non-small cell lung cancer.

Table A2. Development phase results: recall^a^

|  | **N data elements (fields)** | **Recall scores** | | | | |
| --- | --- | --- | --- | --- | --- | --- |
|  |  | **Human** | **Iteration 1** | **Iteration 2** | **Iteration 3** | **Iteration 4** |
| **Study characteristics** | 87 (42) | 0.94 | 0.80 | - | - | - |
| **Baseline characteristics** | 1,077 (170) | 0.96 | 0.94 | - | - | - |
| **Primary efficacy** | 1,449 (149) | 0.97 | 0.82 | Not calculated^c^ | 0.79 | - |
| **Overall efficacy without subgroup** | 6,572 (149) | 0.98 | 0.55 |  | 0.72 | - |
| **Overall efficacy** | 9,587 (149) | 0.97 | Not calculated^b^ |  | 0.58 | - |
| **Safety** | 583 (58) | 0.89 | 0.72 | - | - | - |
| **AEs: NSCLC development** | 2,272 (109) | Iteration 1: 0.81  Iteration 2: 0.73^d^ | 0.46 | 0.57^d^ | - | - |
| **AEs: endometrial cancer development (n=5)** | 5,716 (196) | 0.89 | - | - | General grade:^e^ 0.50 | Specific grade:^f^ 0.71 |

**Footnote:** ^a^ Three NSCLC studies were used unless otherwise stated. ^b^ The prompt did not include a column for subgroup data in this iteration. ^C^ The model failed to consistently retrieve subgroup data (identical prompts applied to the same publications yielded varying results with differing levels of subgroup data extracted), and therefore, a recall score was not calculated for this iteration. ^d^ Only two NSCLC studies were used in this iteration. ^e^ A broad prompt was employed to encourage the model to extract all adverse events of any reported grade. An example of how these AEs might be reported was provided (e.g., 'Grade 3', 'Grade 3-4', or 'any grade'). ^f^ Separate, specific prompts were utilized to encourage the model to extract reported AEs corresponding to each individual grade (e.g., Grade 1), if present in the article. **Abbreviations:** AE, adverse event; NSCLC, non-small cell lung cancer.

Table A3. Testing phase results: precision

|  | **Endometrial Cancer (N=5)** | | | | **HCM (N=5)** | | | |
| --- | --- | --- | --- | --- | --- | --- | --- | --- |
|  | **N data elements (fields)** | **Precision scores** | | | **N data elements (fields)** | **Precision scores** | | |
|  |  | **AI** | **Human** | **Difference** |  | **AI** | **Human** | **Difference** |
| **Study characteristics** | 180 (67) | 0.90 | 0.97 | -0.07 | 156 (50) | 0.98 | 0.99 | -0.01 |
| **Baseline characteristics** | 2,130 (213) | 0.94 | 0.70 | +0.24 | 2,106 (152) | 0.88 | 0.99 | -0.11 |
| **Primary efficacy** | 1,342 (153) | 0.78 | 0.97 | -0.19 | 2,292 (152) | 0.78 | 1.0 | -0.22 |
| **Overall efficacy without subgroup** | 4,326 (153) | 0.25 | 0.95 | -0.7 | 6,727 (152) | 0.25 | 1.0 | -0.75 |
| **Efficacy (overall)** | 8,800 (153) | 0.20 | 0.93 | -0.73 | 12,206 (152) | 0.13 | 1.0 | -0.87 |
| **Safety** | 1,330 (97) | 0.69 | 0.86 | -0.17 | 704 (68) | 0.68 | 0.99 | -0.31 |
| **AEs** | NA^a^ | NA^a^ | NA^a^ | NA^a^ | 2,772 (133) | 0.56 | 1.0 | -0.44 |

**Key: ◼ difference >0 (i.e. AI score was better than the human score); ◼ difference between -0.5 and 0 (i.e. AI score was worse than the human score; ◼ difference ≤-0.5 (i.e. AI score was considerably worse than the human score). Footnotes:** ^a^ The AE prompts were still in development phase when using the endometrial cancer studies and so no test precision score is available. **Abbreviations:** AI, artificial intelligence; AE, adverse event; HCM, hypertrophic cardiomyopathy; NA, not applicable.

Table A4. Testing phase results: recall

|  | **Endometrial Cancer (N=5)** | | | | **HCM (N=5)** | | | |
| --- | --- | --- | --- | --- | --- | --- | --- | --- |
|  | **N data elements (fields)** | **Recall scores** | | | **N data elements (fields)** | **Recall scores** | | |
|  |  | **AI** | **Human** | **Difference** |  | **AI** | **Human** | **Difference** |
| **Study characteristics** | 180 (67) | 0.94 | 0.98 | -0.04 | 156 (50) | 0.78 | 0.97 | -0.19 |
| **Baseline characteristics** | 2,130 (213) | 0.96 | 0.99 | -0.03 | 2,106 (152) | 0.83 | 1.0 | -0.17 |
| **Primary efficacy** | 1,342 (153) | 0.93 | 0.99 | -0.06 | 2,292 (152) | 0.79 | 0.99 | -0.20 |
| **Overall efficacy without subgroup** | 4,326 (153) | 0.92 | 0.85 | +0.07 | 6,727 (152) | 0.75 | 0.99 | -0.24 |
| **Efficacy (overall)** | 8,800 (153) | 0.63 | 0.90 | -0.27 | 12,206 (152) | 0.75 | 0.99 | -0.24 |
| **Safety** | 1,330 (97) | 0.76 | 0.90 | -0.14 | 704 (68) | 0.68 | 0.99 | -0.31 |
| **AEs** | NA^a^ | NA^a^ | NA^a^ | NA^a^ | 2,772 (133) | 0.45 | 1.0 | -0.55 |

**Key: ◼ difference >0 (i.e. AI score was better than the human score); ◼ difference between -0.5 and 0 (i.e. AI score was worse than the human score; ◼ difference ≤-0.5 (i.e. AI score was considerably worse than the human score). Footnotes:** ^a^ The AE prompts were still in development phase when using the endometrial cancer studies and so no test recall score is available. **Abbreviations:** AI, artificial intelligence; AE, adverse event; HCM, hypertrophic cardiomyopathy; NA, not applicable

Table A5. Difference in precision from last development iteration to testing phase

|  | **Final develop-ment set^a^** | **Test set endo-metrial cancer (N=5)** | **Difference vs develop-ment set** | **Test set HCM (N=5)** | **Difference vs develop-ment set** |
| --- | --- | --- | --- | --- | --- |
| **Study characteristics** | 0.93 | 0.90 | -0.03 | 0.98 | +0.05 |
| **Baseline characteristics** | 0.98 | 0.94 | -0.04 | 0.88 | -0.10 |
| **Primary efficacy** | 0.70 | 0.78 | +0.08 | 0.78 | +0.08 |
| **Overall efficacy without subgroup** | 0.21 | 0.25 | +0.04 | 0.25 | +0.04 |
| **Overall efficacy** | 0.19 | 0.20 | +0.01 | 0.13 | -0.06 |
| **Safety** | 0.87 | 0.69 | -0.18 | 0.68 | -0.19 |
| **AEs** | 0.80 | NA^b^ | NA^b^ | 0.56 | -0.24 |

**Key: ◼ difference >0 (i.e. test score was better than the development score); ◼ difference between -0.5 and 0 (i.e. test score was worse than the development score; ◼ difference ≤-0.5 (i.e. test score was considerably worse than the development score). Footnotes:** ^a^ Three NSCLC studies were used in the last development phase for all outcomes except for AE data which used five endometrial studies. ^b^ The AE prompts were still in development phase when using the endometrial cancer studies and so no test precision score is available. **Abbreviations:** AI, artificial intelligence; AE, adverse event; HCM, hypertrophic cardiomyopathy; NA, not applicable.

Table A6. Difference in recall from last development iteration to testing phase

|  | **Final develop-ment set^a^** | **Test set endo-metrial cancer (N=5)** | **Difference vs develop-ment set** | **Test set HCM (N=5)** | **Difference vs develop-ment set** |
| --- | --- | --- | --- | --- | --- |
| **Study characteristics** | 0.80 | 0.94 | +0.14 | 0.78 | -0.02 |
| **Baseline characteristics** | 0.94 | 0.96 | +0.02 | 0.83 | -0.11 |
| **Primary efficacy** | 0.79 | 0.93 | +0.14 | 0.79 | 0 |
| **Overall efficacy without subgroup** | 0.72 | 0.92 | +0.20 | 0.75 | +0.03 |
| **Overall efficacy** | 0.58 | 0.63 | +0.05 | 0.75 | +0.17 |
| **Safety** | 0.72 | 0.76 | +0.04 | 0.68 | -0.04 |
| **AEs** | 0.50 | NA^b^ | NA^b^ | 0.45 | -0.05 |

**Key: ◼ difference >0 (i.e. test score was better than the development score); ◼ difference between -0.5 and 0 (i.e. test score was worse than the development score; ◼ difference ≤-0.5 (i.e. test score was considerably worse than the development score). Footnotes:** ^a^ Three NSCLC studies were used in the last development phase for all outcomes except for AE data which used five endometrial studies. ^b^ The AE prompts were still in development phase when using the endometrial cancer studies and so no test recall score is available. **Abbreviations:** AI, artificial intelligence; AE, adverse event; HCM, hypertrophic cardiomyopathy; NA, not applicable.

Appendix 2: Prompt Sets

1. **Pre-Development Stage**

Pre-development prompts were developed in Microsoft Excel, with one prompt per cell for data extraction. The prompts were fed through a script in Python, which traversed the prompts in Excel and sent the prompt text and relevant article to OpenAI for response generation. The example prompts provided in the embedded document below were derived after five iterations of prompt engineering.

1. **Testing Stage**
   1. Endometrial Cancer
      1. Study Characteristics

*“You are a systematic reviewer investigating clinical outcomes for endometrial cancer. I am going to provide you with some publications which report clinical outcome data associated with endometrial cancer. These publications are all reporting on the same trial. Extract the results from all of these publications into a single table with the following headings: Study name (the first author surname and the year the paper was published e.g. Smith 2023, if the clinical trial has a name i.e., CheckMate 123, report this instead); Study design (state the study design, out of: RCT, Single-arm trial, Prospective cohort, Retrospective cohort, Case-control study, Cross-sectional study. State the term only); Blinding (If the study is an RCT, state whether it is: Open-label, Single-blind or Double-blind. State the term only); Arms: (State whether the trial was composed of a single arm or multiple arms); Single centre/ multicentre (State whether the study was "single-centre" or "multi-centre". State the term only); Geography (select the region/regions which the study was conducted in, choosing from: Asia, North America, Europe, Africa, South America, Australia, Antarctica, International); Countries (list the countries the study was reported in. List alphabetically and separate countries with a comma e.g.: Belgium, UK, US); Study phase (State the study phase, out of: Phase I, Phase II, Phase III, Phase IV. State one answer); Study funding/sponsor (State the type study sponsor or funder, such as industry, academia, medical groups, or governments, as concisely as possible, will probably be reported at the end of the population under Funding); Study funding/sponsor details (Detail the name of the institution/organisation that provided funding for the study); Study start date (State the study start date in dd/mm/yyyy format. State one answer); Number of data cuts (State the number of data cut-offs. State as one number. There will always be a minimum of 1 data cut-off); List data cuts (List the data cut-offs, for example “PFS cut-off”. Separate different data cuts with a comma); Median follow-up for data cuts (provide the median cut-off, in months, for each data cut. Separate values for each data cut with a comma); N screened (State the number of patients screened for the study. State as one number); N enrolled - Entire cohort (State the number of patients enrolled in the study. State as one number); N treated (State the number of patients treated in the study. State as one number); N completed (State the number of patients who completed the study. State as one number); Inclusion criteria (List the inclusion criteria. Separate different points with a semi-colon. Keep the answer concise e.g., do not say "The inclusion criteria were…"); Exclusion criteria (List the exclusion criteria. Separate different points with a semi-colon. Keep the answer concise e.g., do not say "The exclusion criteria were…"); Primary outcome (state the primary outcome); Secondary outcomes (state the secondary outcomes); Duration on treatment (state the duration that patients were treated for in the study. Provide only a single number, the units will be added in the subsequent column); Duration on treatment units (provide the units for the duration on treatment); List of subgroups reported (list the subgroups which data were reported for in the study); Outcomes assessor (State whether outcomes were: investigator-assessed, independently-assessed or both. State the term only); Criteria to evaluate response (State the criteria used to evaluate response. State the term only. For example Response Evaluation Criteria in Solid Tumors [RECIST] version 1.1); Treatment 1 (Follow these steps to identify the first treatment arm and extract the drug name: 1. Locate the section or sentence that describes the treatment arms. 2. Identify the first treatment arm described within this section. 3. Extract the name of the drug or drugs used in this first treatment arm. 4. Provide the drug name or names only, without any additional text. This could be a single word or multiple words if the drug is given in combination with another drug. 5. If no treatment arm is described or the drug name or names are not reported, respond with "NR". For example: If the text states "Patients were randomized 1:1 to receive oral apatinib 500 mg and gefitinib 250 mg (A + G group) or oral placebo and gefitinib 250 mg (P + G group) once per day for a 28-day cycle", the answer should be "Apatinib and gefitinib". If no information about the first treatment arm is provided, respond with "NR"); Regimen for treatment 1 (State the dosing schedule and administration for the first treatment arm. Use the following format: Drug name, X mg/kg, frequency (e.g., once per day; every 3 weeks etc. intravenous or oral tablet); Number of patients in treatment arm 1 (state how many patients were allocated to receive treatment 1); Treatment 2 (Follow these steps to identify the second treatment arm and extract the drug name: 1. Locate the section or sentence that describes the treatment arms. 2. Identify the second treatment arm described within this section. 3. Extract the name of the drug or drugs used in this second treatment arm. 4. Provide the drug name or names only, without any additional text. This could be a single word or multiple words if the drug is given in combination with another drug. 5. If no treatment arm is described or the drug name or names are not reported, respond with "NR". For example: If the text states "Patients were randomized 1:1 to receive oral apatinib 500 mg and gefitinib 250 mg (A + G group) or oral placebo and gefitinib 250 mg (P + G group) once per day for a 28-day cycle", the answer should be "Placebo and gefitinib". If no information about the second treatment arm is provided, respond with "NR"); Regimen for treatment 2 (State the dosing schedule and administration for the first treatment arm. Use the following format: Drug name, X mg/kg, frequency (e.g., once per day; every 3 weeks etc. intravenous or oral tablet); Number of patients in treatment arm 2 (state how many patients were allocated to receive treatment 2); Treatment 3 (Follow these steps to identify the third treatment arm and extract the drug name: 1. Locate the section or sentence that describes the treatment arms. 2. Identify the second treatment arm described within this section. 3. Extract the name of the drug or drugs used in this third treatment arm. 4. Provide the drug name or names only, without any additional text. This could be a single word or multiple words if the drug is given in combination with another drug. 5. If no treatment arm is described or the drug name or names are not reported, respond with "NR". For example: If the text states "Patients were randomized 1:1 to receive oral apatinib 500 mg and gefitinib 250 mg (A + G group) or oral placebo and gefitinib 250 mg (P + G group) once per day for a 28-day cycle", the answer should be "Placebo and gefitinib". If no information about the second treatment arm is provided, respond with "NR"); Regimen for treatment 3 (State the dosing schedule and administration for the third treatment arm. Use the following format: Drug name, X mg/kg, frequency (e.g., once per day; every 3 weeks etc. intravenous or oral tablet); Number of patients in treatment arm 3 (state how many patients were allocated to receive treatment 3). If any information is not available, enter NR. Double check that all results have been added to this table from the paper. If anything is missing, add it to the table. Check for any mistakes.”*

**Next sub-prompt** (If multiple publications):

*“I am now going to provide some more publications reporting on the same study. Add any additional information from these publications to the table you just produced.”*

- - 1. Baseline Characteristics

*“You are a systematic reviewer investigating clinical outcomes for endometrial cancer. I am going to provide you with some publications which reports clinical outcome data associated with endometrial cancer. These publications are all reporting on the same trial. Extract the results from all of these publications into a table. Make a new row for each treatment arm identified in the publication, and report the corresponding data in the relevant row. For example, the information and results for treatment arm 1 “Apatinib and gefitinib" will be reported on row one and the information and results for treatment arm 2 “Placebo” will be reported in the second row. Follow these steps to identify the treatment arms 1. Locate the section or sentence that describes the treatment arms. 2. Identify the different treatment arms described within this section with the first treatment arm described first and second treatment arm described second. For example, if the text states "Patients were randomized 1:1 to receive oral apatinib 500 mg and gefitinib 250 mg (A + G group) or oral placebo and gefitinib 250 mg (P + G group) once per day for a 28-day cycle", the first treatment group should be "Apatinib and gefitinib" and the second treatment group “placebo and gefitinib”. For the table use the following headings: Study name (the first author surname and the year the paper was published e.g. Smith 2023, if the clinical trial has a name i.e. CheckMate 123,report this instead); Study setting (State the study design of the publication, for example “RCT”, “observational study” etc); Treatment arm (Extract the drug names for each treatment arm); Subgroup name (If data is reported for the entire cohort, put “All” and if the data is reported by subgroups, specify the subgroup); Line of treatment (state first line as 1L, second line as 2L etc); Treatment category (Extract the category of the primary interventions under investigation. For example, Chemotherapy, PD-1 inhibitors, protein kinase inhibitors. Separate different interventions with a comma); Treatment dose and regimen (very concisely state the treatment dose and regimen for this treatment arm, use + and or for combinations); N assigned to arm (State the sample size for the entire cohort. State as one number); Sample size (State the sample size for the number of people assigned to the relevant treatment arm. State as one number); Analysis set (Choose from “intention-to-treat”, “per protocol”, “modified intention-to-treat” or “safety set”); Details of analysis set (Extract key details for the analysis set, for example “all randomised patients”, “patients with outcome data from at least one follow-up assessment” etc). For frequency, use appropriate notation (e.g., QD for once daily, BID for twice a day, QID for four times a day, etc.); Reported? –age (Is information on age reported for each treatment arm? If yes, state "Y", if no, state "NR"); Mean – age (state the mean age. State as one number without units. E.g., if mean age is 65 years, the answer should be "65". For example: If the text states "Age, mean (range) 55 (40-84)", the answer should be "55" but if the text states "Age, median (range) 55 (40-84)", the answer should be "NR"); Median – age (state the median age. State as one number without units); SD – age (state the standard deviation [SD] for age. State as one number); SE – age (State the standard error [SE] for age. State as one number); IQR Q1 – age (State the lower inter-quartile range [IQR] for age. For example: If the text states "The inter-quartile range [IQR] for age 51–66” the answer should be "51”). IQR Q3– age (State the upper IQR for age. For example: If the text states " inter-quartile range [IQR] for age 51–66” the answer should be "66”). Range lower limit – age (State the lower range value for age. State as one number. For example: If the text states "Age, median (range), years 60 (27 to 78).", the answer should be "27".); Range upper limit – age (State the upper range value for age. State as one number. For example: If the text states "Age, median (range), years 60 (27 to 78)" the answer should be "78"); Denominator-age (state the number of patients who were included in the calculations for age); p vs comparator – age (State the p value for the treatment groups for age. State as one number." For example, if the text states "Age, median (range), years 60 (27 to 78), p value 0.3", the answer should be "0.3".); Specify comparator- age (If a p value for the treatment arm compared to a comparator for age is reported, report the comparator. Be as concise as possible by stating only the name of the comparator group e.g. "Docetaxel"); Reported as categorical- age (if the data is reported in categories e.g. >75 years old, report “Y”). For any information not reported in the publication, respond with "NR". For any numerical values, state as one number and report any decimals places. Double check that all relevant data have been added to this table from the paper. If anything is missing, add it to the table. Check for any mistakes such as adding median data into cells which should only report the mean.”*

**Next sub-prompt:**

*“Now make a table for sex and ethnicity results. As with the table above, make a new row for each treatment arm identified in the publication, and report the corresponding data in the relevant row. For the table use the following headings: Study name (the first author surname and the year the paper was published e.g. Smith 2023, if the clinical trial has a name i.e. CheckMate 123,report this instead); Treatment arm (Extract the drug names for each treatment arm); Reported? – race or ethnicity (If information on race or ethnicity is reported for the treatment arm, state "Y"); n, white – race or ethnicity (state the number of white patients); %, white – race or ethnicity (state the percentage of white patients); n, black – race or ethnicity (state the number of black patients); %, black – race or ethnicity (state the percentage of black patients. State as one number.); n, Hispanic – race or ethnicity (state the number of Hispanic patients.); %, Hispanic – race or ethnicity (state the percentage of Hispanic patients); n, Asian – race or ethnicity (state the number of Asian patients); %, Asian – race or ethnicity (state the percentage of Asian patients); n, other – race or ethnicity (, state the number of patients listed as having "Other" race or ethnicity.); %, other – race or ethnicity (state the percentage of patients listed as having "Other" race or ethnicity.); n, unknown – race or ethnicity (state the number of patients listed as having "Unknown" race or ethnicity. ); %, unknown – race or ethnicity (state the percentage of patients listed as having "Unknown" race or ethnicity); Denominator – race or ethnicity (state the number of patients who were included in the calculations for race or ethnicity); p vs comparator – race or ethnicity (For example, if the text states "Race, P 0.480", the answer should be "0.480".); Specify comparator- race or ethnicity (If a p value for the treatment arm compared to a comparator for race or ethnicity is reported, report the comparator. Be as concise as possible by stating only the name of the comparator group e.g. "Docetaxel"); Reported? – geographic region (If information on geographic region of the patients is reported for the treatment arms, state “Y”); n, Asia – geographic region (state the number of patients from Asia); %, Asia – geographic region (state the percentage of patients from Asia); n, Europe – geographic region (state the number of patients from Europe); %, Europe – geographic region (state the percentage of patients from Europe); n, North America– geographic region (state the number of patients from North America); %, North America – geographic region (state the percentage of patients from North America); n, South America– geographic region (state the number of patients from South America); %, South America – geographic region (state the percentage of patients from South America); n, Australasia – geographic region (state the number of patients from Australasia); %, Australasia – geographic region (state the percentage of patients from Australasia); n, Africa – geographic region (state the number of patients from Africa); %, Africa – geographic region (state the percentage of patients from Africa); Denominator – geographic region (state the number of patients who were included in the calculations for geographic region); p vs comparator – geographic region (For example, if the text states "Race, P 0.480", the answer should be "0.480".); Specify comparator- geographic region (If a p value for the treatment arm compared to a comparator for race or ethnicity is reported, report the comparator. Be as concise as possible by stating only the name of the comparator group e.g. "Docetaxel"). For any information not reported in the publication, respond with "NR". For any numerical values, state as one number and report any decimals places. Can you double check that all relevant data have been added to this table from the paper. Check for any mistakes such as adding median data into cells which should only report the mean.”*

**Next sub-prompt:**

*“Now make a table for disease staging and performance status results. As with the table above, make a new row for each treatment arm identified in the publication, and report the corresponding data in the relevant row. For the table use the following headings: Study name (the first author surname and the year the paper was published e.g. Smith 2023, if the clinical trial has a name i.e. CheckMate 123,report this instead); Treatment arm (Extract the drug names for each treatment arm); Reported? –disease staging (Is information on disease staging reported for each treatment arm? If yes, state "Y", if no, state "NR"); n, Stage IIIA – disease staging (state the number of patients with Stage IIIA (or 3a) cancer); %, Stage IIIA – disease staging (state the number of patients with Stage IIIA (or 3a) cancer); n, Stage IIIB – disease staging (state the number of patients with Stage IIIB (or 3b) cancer.); %, Stage IIIB – disease staging (state the percentage of patients with Stage IIIB (or 3b) cancer); n, Stage IIIC – disease staging (state the number of patients with Stage IIIC (or 3c) cancer); %, Stage IIIC – disease staging (state the number of patients with Stage IIIC (or 3c) cancer); n, Stage IIIC1 – disease staging (state the number of patients with Stage IIIC1 (or 3c1) cancer); %, Stage IIIC1 – disease staging (state the number of patients with Stage IIIC1 (or 3c1) cancer); n, Stage IIIC12– disease staging (state the number of patients with Stage IIIC2 (or 3c2) cancer); %, Stage IIIC2 – disease staging (state the number of patients with Stage IIIC2 (or 3c2) cancer); n, Stage III – disease staging (state the number of patients with Stage III (or 3) cancer.); %, Stage III – disease staging (state the percentage of patients with Stage III (or 3) cancer); n, Stage IV – disease staging (state the number of patients with Stage IV (or 4) cancer.); %, Stage IV – disease staging (state the percentage of patients with Stage IV (or 4) cancer); n, newly diagnosed – disease staging (state the number of patients with a recent diagnosis of cancer.); %, newly diagnosed – disease staging (state the percentage of patients with a recent diagnosis of cancer); n, recurrent disease – disease staging (state the number of patients with recurrent cancer); %, recurrent disease – disease staging (state the percentage of patients with recurrent cancer); Denominator – disease staging (state the number of patients who were included in the calculations for disease staging); p vs comparator – disease staging (For example, if the text states "disease status, P 0.480", the answer should be "0.480"); Specify comparator- disease staging (If a p value for the treatment arm compared to a comparator for disease status is reported, report the comparator. Be as concise as possible by stating only the name of the comparator group e.g. "Docetaxel"); Overlapping categories- disease staging (if the data is reported in overlapping categories e.g. IIIC/IV, report “Y”). Reported? –performance status (Is information on performance status reported for each treatment arm? If yes, state "Y", if no, state "NR"); Scale – performance status (What scale was used to assess performance? For example, it could be ECOG, WHO etc); n, 0 – performance status (state the number patients with performance status [PS] of 0); %, 0 – performance status (state the percentage of patients with PS of 0); n, 1 – performance status (state the number of patients with PS of 1); %, 1 – performance status (state the percentage of patients with PS of 1); n, 2 – performance status (state the number of patients with PS of 2); %, 2 – performance status (state the percentage of patients with PS of 2); n, 3 – performance status (state the number of patients with PS of 3); %, 3 – performance status (state the percentage of patients with PS of 3); n, 4 – performance status (state the number of patients with PS of 4); %, 4 – performance status (state the percentage of patients with PS of 4.); Denominator – performance status (state the number of patients who were included in the calculations for performance status); p vs comparator – performance status (For example, if the text states "performance status, P 0.480", the answer should be "0.480"); Specify comparator- performance status (If a p value for the treatment arm compared to a comparator for performance status is reported, report the comparator. Be as concise as possible by stating only the name of the comparator group e.g. "Docetaxel"); Reported as categorical- performance status (if the data is reported in categories e.g. ECOG status >2, report “Y”). For any information not reported in the publication, respond with "NR". For any numerical values, state as one number and report any decimals places. Double check that all relevant data have been added to this table from the paper. If anything is missing, add it to the table. Check for any mistakes such as adding median data into cells which should only report the mean.”*

**Next sub-prompt:**

*“Now make a table for previous types of therapies and previous lines of therapies results. As with the table above, make a new row for each treatment arm identified in the publication, and report the corresponding data in the relevant row. For the table use the following headings: Study name (the first author surname and the year the paper was published e.g. Smith 2023, if the clinical trial has a name i.e. CheckMate 123,report this instead); Treatment arm (Extract the drug names for each treatment arm); Reported? –previous type of therapy (Is information on the types of treatments received by patients before the study reported for each treatment arm? If yes, state "Y", if no, state "NR"); n, prior radiotherapy – previous type of therapy (state the number of patients who received radiotherapy before the study); %, prior radiotherapy – previous type of therapy (state the percentage of patients who received radiotherapy before the study); n, prior surgery – previous type of therapy (state the number of patients who received surgery before the study); %, prior surgery – previous type of therapy (state the percentage of patients who received surgery before the study); Definition, neoadjuvant therapy – previous type of therapy (If patients received neoadjuvant therapy, extract the definition of neoadjuvant if provided by the authors); n, neoadjuvant therapy – previous type of therapy (state the number of patients who received neoadjuvant therapy before the study); %, neoadjuvant therapy – previous type of therapy (state the percentage of patients who received neoadjuvant therapy before the study); Definition, adjuvant therapy – previous type of therapy (If patients received adjuvant therapy, extract the definition of adjuvant if provided by the authors); n, adjuvant therapy – previous type of therapy (state the number of patients who received adjuvant therapy before the study); %, adjuvant therapy – previous type of therapy (state the percentage of patients who received adjuvant therapy before the study); Denominator – previous type of therapy (state the number of patients who were included in the calculations for previous types of therapies); p vs comparator – previous type of therapy (For example, if the text states "previous therapy type, P 0.480", the answer should be "0.480"); Specify comparator- previous therapy type (If a p value for the treatment arm compared to a comparator for previous types of therapy is reported, report the comparator. Be as concise as possible by stating only the name of the comparator group e.g. "Docetaxel"). Reported? –prior lines of therapy (Is information on treatment line present for the treatment arm? If yes, state "Y", if no, state "NR"); Definition – prior lines of therapy (Extract details of if treatment is in the metastatic setting or adjuvant/neoadjuvant setting); n, 0 – prior lines of therapy (state the number of patients who have received no prior treatment); %, 0 – prior lines of therapy (state the percentage of patients who have received no prior treatment); n, 1– prior lines of therapy (state the number of patients who have received 1 prior line of treatment); %, 1 – prior lines of therapy (state the percentage of patients who have received 1 prior line of treatment); Denominator – prior lines of therapy (state the number of patients who were included in the calculations for prior lines of therapy); p vs comparator – prior lines of therapy (For example, if the text states "1 prior line of therapy, P 0.480", the answer should be "0.480"); Specify comparator- prior lines of therapy (If a p value for the treatment arm compared to a comparator for a different prior therapy lines is reported, report the comparator. Be as concise as possible by stating only the name of the comparator group e.g. "Docetaxel").check for any mistakes such as adding percentages into the n-cells.”*

**Next sub-prompt:**

*“Now make a table for histology subtype and programmed cell death ligand 1 (PD-L1) expression results. As with the table above, make a new row for each treatment arm identified in the publication, and report the corresponding data in the relevant row. For the table use the following headings: Study name (the first author surname and the year the paper was published e.g. Smith 2023, if the clinical trial has a name i.e. CheckMate 123,report this instead); Treatment arm (Extract the drug names for each treatment arm); Reported? –histology subtype (is information on cancer histology subtype reported for each treatment arm? If yes, state "Y", if no, state "NR"); n, serous carcinoma-histology subtype (state the number of patients with serous carcinoma); %, serous carcinoma-histology subtype (state the percentage of patients with serous adenocarcinoma); n, serous adenocarcinoma -histology subtype (state the number of patients with large cell carcinoma); %, serous adenocarcinoma -histology subtype (state the percentage of patients with serous adenocarcinoma); n, clear cell carcinoma-histology subtype (state the number of patients with clear cell carcinoma); %, clear cell carcinoma-histology subtype (state the percentage of patients with clear cell carcinoma); n, clear cell adenocarcinoma-histology subtype (state the number of patients with clear cell adenocarcinoma); %, endometrioid carcinoma-histology subtype (state the percentage of patients with endometrioid carcinoma); n, endometrioid carcinoma-histology subtype (state the number of patients with endometrioid carcinoma); %, endometrioid carcinoma-histology subtype (state the percentage of patients with endometrioid carcinoma); n, endometrioid adenocarcinoma-histology subtype (state the number of patients with endometrioid adenocarcinoma); %, endometrioid adenocarcinoma-histology subtype (state the percentage of patients endometrioid adenocarcinoma); n, mucinous carcinoma-histology subtype (state the number of patients with mucinous carcinoma); %, mucinous carcinoma-histology subtype (state the percentage of patients with mucinous carcinoma); n, mucinous adenocarcinoma-histology subtype (state the number of patients with mucinous adenocarcinoma); %, mucinous adenocarcinoma-histology subtype (state the percentage of patients with mucinous adenocarcinoma); n, transitional cell carcinoma-histology subtype (state the number of patients with transitional cell carcinoma); %, transitional cell carcinoma-histology subtype (state the percentage of patients with transitional cell carcinoma); n, squamous cell carcinoma-histology subtype (state the number of patients with squamous cell carcinoma); %, squamous cell carcinoma-histology subtype (state the percentage of patients with squamous cell carcinoma); n, carcinosarcoma-histology subtype (state the number of patients with carcinosarcoma); %, carcinosarcoma-histology subtype (state the percentage of patients with carcinosarcoma); n, mixed epithelial-histology subtype (state the number of patients with mixed epithelial); %, mixed epithelial-histology subtype (state the percentage of patients mixed epithelial); n, unspecified-histology subtype (state the number of patients with unspecified); %, unspecified-histology subtype (state the percentage of patients with unspecified); n, undifferentiated-histology subtype (state the number of patients with undifferentiated); %, undifferentiated-histology subtype (state the percentage of patients with undifferentiated); n, other-histology subtype (state the number of patients with other); %, other-histology subtype (state the percentage of patients with other); Denominator – histology subtype (state the number of patients who were included in the calculations for histology subtype); p vs comparator – histology subtype (For example, if the text states "adenocarcinoma, P 0.480", the answer should be "0.480"); Specify comparator- histology subtype (If a p value for the treatment arm compared to a comparator for a different histology subtype is reported, report the comparator. Be as concise as possible by stating only the name of the comparator group e.g. "Docetaxel"). Reported? –PD-LI expression (Is information on PD-L1 expression present for the treatment arm? If yes, state "Y", if no, state "NR"); Diagnosis method-PD-L1 (Extract the method used for diagnosing PD-L1 expressions); n, positive -PD-L1 expression (state the number of patients who are PD-L1 positive); %, positive – PD-L1 expression (state the percentage of patients who are PD-L1 positive); n, negative -PD-L1 (state the number of patients who are PD-L1 negative); %, negative – PD-L1 expression (state the percentage of patients who are PD-L1 negative); Definition, high-grade-PD-L1 expression (Extract the definition of high-grade PD-L1 expression used); n, high-grade-PD-L1 expression (state the number of patients who are high grade for PD-L1 expression); %, high-grade-PD-L1 expression (state the percentage of patients who are high grade for PD-L1 expression); Definition, low-grade-PD-L1 expression (Extract the definition of low-grade PD-L1 expression used); n, low-grade-PD-L1 expression (state the number of patients who are low-grade for PD-L1 expression); %, low-grade-PD-L1 expression (state the percentage of patients who are low-grade for PD-L1 expression); n, unknown-PD-L1 expression (state the number of patients who have unknown information on PD-L1 expression); %, unknown-PD-L1 expression (state the percentage of patients who have unknown information on PD-L1 expression); Denominator – PD-L1 expression (state the number of patients who were included in the calculations for PD-L1 expression); p vs comparator – PD-L1 expression (For example, if the text states "PD-L1 positive, P 0.480", the answer should be "0.480"); Specify comparator- PD-L1 expression (If a p value for the treatment arm compared to a comparator for a different PD-L1 expression is reported, report the comparator. Be as concise as possible by stating only the name of the comparator group e.g. "Docetaxel").check for any mistakes such as adding percentages into the n-cells.”*

**Next sub-prompt:**

*“Now make a table for human epidermal growth factor receptor 2 (HER2) status results. As with the table above, make a new row for each treatment arm identified in the publication, and report the corresponding data in the relevant row. For the table use the following headings: Study name (the first author surname and the year the paper was published e.g. Smith 2023, if the clinical trial has a name i.e. CheckMate 123,report this instead); Treatment arm (Extract the drug names for each treatment arm); Reported? – HER2 status (is information on HER2 status reported for each treatment arm? If yes, state "Y", if no, state "NR"); Diagnosis method-HER2 status (Extract the method used for diagnosing HER2 status); n, positive -HER2 status (state the number of patients who are HER2 positive); %, positive – HER2 status (state the percentage of patients who are HER2 positive); n, negative - HER2 status (state the number of patients who are HER2 negative); %, negative – HER2 status (state the percentage of patients who are HER2 negative); Denominator – HER2 status (state the number of patients who were included in the calculations for HER2 status); p vs comparator – HER2 status (For example, if the text states "HER2 positive, P 0.480", the answer should be "0.480"); Specify comparator- HER2 status (If a p value for the treatment arm compared to a comparator for a different HER2 status is reported, report the comparator. Be as concise as possible by stating only the name of the comparator group e.g. "Docetaxel").”*

**Next sub-prompt:**

*“Now make a table for mismatch repair (MMR) or microsatellite stable (MSS) status results. MMR and MSS maybe be used interchangeably in studies, to extract any relevant data. As with the table above, make a new row for each treatment arm identified in the publication, and report the corresponding data in the relevant row. For the table use the following headings: Study name (the first author surname and the year the paper was published e.g. Smith 2023, if the clinical trial has a name i.e. CheckMate 123,report this instead); Treatment arm (Extract the drug names for each treatment arm); Reported? – MMR/MSS status (is information on MMR or MSS status reported for each treatment arm? If yes, state "Y", if no, state "NR"); Diagnosis method-MMR/MSS status (Extract the method used for diagnosing MMR/MSS status); n, mismatch repair proficient - MMR/MSS status (state the number of patients who are mismatch repair proficient); %, mismatch repair proficient – MMR/MSS status (state the percentage of patients who are mismatch repair proficient); n, mismatch repair deficient - MMR/MSS status (state the number of patients who are mismatch repair deficient); %, mismatch repair deficient – MMR/MSS status (state the percentage of patients who are mismatch repair deficient); n, MSS high - MMR/MSS status (state the number of patients who are MSS high); %, MSS high– MMR/MSS status (state the percentage of patients who are MSS high); n, MSS stable- MMR/MSS status (state the number of patients who are MSS stable); %, MSS stable– MMR/MSS status (state the percentage of patients who are MSS stable); Denominator – MMR/MSS status (state the number of patients who were included in the calculations for MMR/MSS status); p vs comparator – MMR/MSS status (For example, if the text states "mismatch repair deficient , P 0.480", the answer should be "0.480"); Specify comparator- MMR/MSS (If a p value for the treatment arm compared to a comparator for a different MMR/MSS status is reported, report the comparator. Be as concise as possible by stating only the name of the comparator group e.g. "Docetaxel").”*

**Next sub-prompt:**

*“Now make a table for Breast Cancer (BRCA) and homologous recombination repair (HRR) gene mutation status results. As with the table above, make a new row for each treatment arm identified in the publication, and report the corresponding data in the relevant row. For the table use the following headings: Study name (the first author surname and the year the paper was published e.g. Smith 2023, if the clinical trial has a name i.e. CheckMate 123,report this instead); Treatment arm (Extract the drug names for each treatment arm); Reported? – BRCA status (is information on BRCA status reported for each treatment arm? If yes, state "Y", if no, state "NR"); Diagnosis method-BRCA status (Extract the method used for diagnosing BRCA status); n, mutant-BRCA status (state the number of patients who are have a BRCA mutant status); %, mutant-BRCA status (state the percentage of patients who are have a BRCA mutant status); n, wild-type-BRCA status (state the number of patients who are have a BRCA wildtype status); %, wild-type-BRCA status (state the percentage of patients who are have a BRCA wild-type status); n, unknown –BRCA status (state the number of patients who have a unknown BRCA status); %, unknown - BRCA status (state the percentage of patients who have a unknown BRCA status); Denominator – BRCA status (state the number of patients who were included in the calculations for BRCA status); p vs comparator – BRCA status (For example, if the text states "mutant, P 0.480", the answer should be "0.480"); Specify comparator- BRCA status (If a p value for the treatment arm compared to a comparator for a different BRCA status is reported, report the comparator. Be as concise as possible by stating only the name of the comparator group e.g. "Docetaxel"). Reported? – HRR status (is information on HRR status reported for each treatment arm? If yes, state "Y", if no, state "NR"); Diagnosis method-HRR status (Extract the method used for diagnosing HRR status); n, mutant-HRR status (state the number of patients who are have a HRR mutant status); %, mutant-HRR status (state the percentage of patients who are have a HRR mutant status); n, wild-type-HRR status (state the number of patients who are have a HRR wildtype status); %, wild-type-HRR status (state the percentage of patients who are have a HRR wild-type status); n, unknown –HRR status (state the number of patients who have a unknown HRR status); %, unknown - HRR status (state the percentage of patients who have a unknown HRR status); Denominator – HRR status (state the number of patients who were included in the calculations for HRR status); p vs comparator – HRR status (For example, if the text states "mutant, P 0.480", the answer should be "0.480"); Specify comparator- HRR status (If a p value for the treatment arm compared to a comparator for a different HRR status is reported, report the comparator. Be as concise as possible by stating only the name of the comparator group e.g. "Docetaxel").”*

- - 1. Efficacy

*“You are a systematic reviewer investigating clinical outcomes for endometrial cancer. I will provide you with publications and their supplementary materials that report clinical outcome data associated with endometrial cancer. Extract the results from these publications and their supplementary materials, ensuring to include all possible subgroups, particularly those documented in figures and tables, from all relevant sections, figures, and tables into one comprehensive table. Separate the following onto new rows: different treatment arms. Follow these steps:*

*1. Identify Treatment Arms:*

*Locate the section or sentence that describes the treatment arms.*

*Identify the different treatment arms described within this section with the first treatment arm described first and the second treatment arm described second. For example, if the text states "Patients were randomized 1:1 to receive oral apatinib 500 mg and gefitinib 250 mg (A + G group) or oral placebo and gefitinib 250 mg (P + G group) once per day for a 28-day cycle", the first treatment group should be "Apatinib and gefitinib" and the second treatment group “Placebo and gefitinib”.*

*2. Table Formatting:*

*Use the specified headings: Study name (the first author surname and the year the paper was published e.g. Smith 2023, if the clinical trial has a name i.e. CheckMate 123,report this instead), Treatment arm (Extract the drug names for each treatment arm), Treatment line (state first line as 1L, second line as 2L etc), Treatment category (state as platinum chemotherapy, non-platinum chemotherapy, placebo, non-chemotherapy, non-chemotherapy plus chemotherapy), Subgroup name, Analysis set (State the analysis set out of: Intention to treat [ITT], Modified intention to treat [mITT], Per protocol [PP], subgroups. State the term only), Details of analysis set (concisely state details of the analysis set e.g. “All patients randomised to the standard arm with at least one known treatment administration”), Outcome assessor (state how outcomes were assessed in the study out of: "Investigator" or "Independent/central review". State the term only. For example, if the text states "Outcomes were assessed by investigators.", the answer should be "Investigator"), Assessment criteria (State the assessment criteria used to evaluate response. State the term only. For example if the text states "The antitumor response was assessed by Response Evaluation Criteria in Solid Tumors [RECIST] version 1.1.", the answer should be "Response Evaluation Criteria in Solid Tumors (RECIST) version 1.1), Type of timepoint (state the type of timepoint(s) at which outcomes are reported for the treatment arm, for example if a specific date or time is used, like "Month 6" or "01/01/2018" - state “Specific timepoint”. If data is presented after follow-up without a specific timepoint reported, state “Data cut-off/follow-up” only use these two options), Timepoint (State the timepoint(s) at which outcomes are reported for the treatment arm, for example this might look like "Month 6" or a specific date like "01/01/2018" - state only the timepoint. If data is presented after follow-up without a specific timepoint reported, state 'Data cut-off'. For example: If the study reports a median or mean value for an outcome (e.g. median PFS), with data presented after follow-up, the answer should be 'Data cut-off' or a specific data e.g. "01/01/2016" if the date of cut-off is presented. If the study reports a survival outcome at a specific timepoint e.g. 6-month progression free survival (PFS), the answer should be "Month 6". If the study reports a survival outcome at another timepoint e.g. 1-year PFS, report “Year 1” on another row), Date of cut-off (If “Data cut-off/follow-up” is stated in the type of timepoint heading, state the date of the cut-off in this format DD/MM/YYYY. For example if the text states “At data cutoff (January 15, 2020) median follow-up was” report 15/01/2020), Median follow-up (State the median follow-up time, for the treatment arm. Do not state the units. State only the numerical value e.g. "14.2"), Unit – follow-up (state the units of the median follow-up e.g. months), Reported? –PFS (If PFS is reported for this row, state "Y"), Definition -PFS, Denominator -PFS, n, PFS-PFS, %, PFS -PFS, Units- PFS (State the unit used for PFS, out of "days", "weeks", "months" or "years". State the unit only), Median -PFS, Mean -PFS, SE- PFS, SD -PFS, IQR, Q1-PFS, IQR, Q3 -PFS, 95% CI, lower -PFS, 95% CI, upper -PFS, p vs baseline -PFS, p vs comparator-PFS, Specify comparator- PFS (If a p value for the treatment arm compared to a comparator for PFS is reported, report the comparator. Be as concise as possible by stating only the name of the comparator group e.g. "Docetaxel". If this treatment arm and timepoint is not statistically compared to a comparator for PFS, or if a p-value is only presented for the hazard ratio (HR), state "NR"), HR, OR, RR reported? -PFS (If HR, odds ratio [OR] or risk ratio [RR] is reported for PFS state the corresponding abbreviation e.g. “HR”), Point estimate, HR/OR/RR- PFS , SE, HR/OR/RR - 95% CI, lower bound, HR -PFS, 95% CI, upper bound, HR -PFS, p vs comparator, HR-PFS, Specify comparator, HR -PFS (if a p value for the HR, OR or RR for this treatment arm and timepoint compared to a comparator for PFS is reported, report the comparator. The comparator must be different to the treatment arm. Be as concise as possible e.g. "Docetaxel"), n, responders-PFS, %, responders-PFS, n, unmeasurable -PFS (State the number of patients with unmeasurable PFS. Could be reported as “unmeasurable” or “not evaluable”), %, unmeasurable -PFS (State the percentage of patients with unmeasurable PFS. Could be reported as “unmeasurable” or “not evaluable”), n, unknown -PFS, %, unknown -PFS*

*3. Ensure Completeness and Specificity:*

*Double-check that all PFS data and subgroup analyses from the entire documents are thoroughly represented in the table, with separate rows for each treatment arm as needed.*

*If any information is not reported, respond with "NR". Be meticulous in including all data in the figures and tables in the table to ensure comprehensiveness.”*

**Next sub-prompt:**

*“Now make table rows, using the same headings and format for different timepoints. A table is not needed if there are no additional data.”*

**Next sub-prompt:**

*“Now make table rows, using the same headings and format for different assessors so that “Investigator" or "Independent/central review" are extracted if needed. A table is not needed if there are no additional data.”*

**Next sub-prompt:**

*“Now make table rows, using the same headings for different analysis sets. A table is not needed if there are no additional data.”*

**Next sub-prompt:**

*“Now identify all possible subgroups reported across the entirety of the publications, including supplementary tables and figures, to ensure comprehensive representation. Examples of potential subgroups include: gender (male or female), age, ECOG performance status, smoking status, severity of disease, tumour stage, mutation status, prior lines of therapy, race. Make rows, using the same headings for all identified subgroups. A table is not needed if there is no additional data.”*

**Next sub-prompt:**

*“Now make the same original table, but for overall survival (OS) instead of PFS.”*

**Next sub-prompt:**

*“Now make rows, using the same headings for different timepoints for OS. A table is not needed if there are no additional data.”*

**Next sub-prompt:**

*“Now make rows, using the same headings for different assessors for OS. A table is not needed if there are no additional data.”*

**Next sub-prompt:**

*“Now make rows, using the same headings for different analysis sets for OS. A table is not needed if there are no additional data.”*

**Next sub-prompt:**

*“Now identify all possible subgroups reported across the entirety of the publications, including supplementary tables and figures, to ensure comprehensive representation. Examples of potential subgroups include: gender (male or female), age, ECOG performance status, smoking status, severity of disease, tumour stage, mutation status, PD-L1 status. Make rows, using the same headings for all identified subgroups for OS. A table is not needed if there are no additional data.”*

**Next sub-prompt:**

*“Now make the same original table, but for overall response rate (ORR) instead of PFS.”*

**Next sub-prompt:**

*“Now make rows, using the same headings for different timepoints for ORR. A table is not needed if there are no additional data.”*

**Next sub-prompt:**

*“Now make rows, using the same headings for different assessors for ORR. A table is not needed if there are no additional data.”*

**Next sub-prompt:**

*“Now make rows, using the same headings for different analysis sets for ORR. A table is not needed if there are no additional data.”*

**Next sub-prompt:**

*“Now identify all possible subgroups reported across the entirety of the publications, including supplementary tables and figures, to ensure comprehensive representation. Examples of potential subgroups include: gender (male or female), age, ECOG performance status, smoking status, severity of disease, tumour stage, mutation status, prior lines of therapy, race. Make rows, using the same headings for all identified subgroups for ORR. A table is not needed if there are no additional data.”*

**Next sub-prompt:**

*“Now make the same original table, but for duration of response (DOR) instead of PFS.”*

**Next sub-prompt:**

*“Now make rows, using the same headings for different timepoints for DOR. A table is not needed if there are no additional data.”*

**Next sub-prompt:**

*“Now make rows, using the same headings for different assessors for DOR. A table is not needed if there are no additional data.”*

**Next sub-prompt:**

*“Now make rows, using the same headings for different analysis sets for DOR. A table is not needed if there are no additional data.”*

**Next sub-prompt:**

*“Now identify all possible subgroups reported across the entirety of the publications, including supplementary tables and figures, to ensure comprehensive representation. Examples of potential subgroups include: gender (male or female), age, ECOG performance status, smoking status, severity of disease, tumour stage, mutation status, prior lines of therapy, race. Make rows, using the same headings for all identified subgroups for DOR. A table is not needed if there are no additional data.”*

**Next sub-prompt:**

*“Now make the same original table, but for time to subsequent therapy (TTST) instead of PFS.”*

**Next sub-prompt:**

*“Now make rows, using the same headings for different timepoints for TTST. A table is not needed if there are no additional data.”*

**Next sub-prompt:**

*“Now make rows, using the same headings for different assessors for TTST. A table is not needed if there are no additional data.”*

**Next sub-prompt:**

*“Now make rows, using the same headings for different analysis sets for TTST. A table is not needed if there are no additional data.”*

**Next sub-prompt:**

*“Now identify all possible subgroups reported across the entirety of the publications, including supplementary tables and figures, to ensure comprehensive representation. Examples of potential subgroups include: gender (male or female), age, ECOG performance status, smoking status, severity of disease, tumour stage, mutation status, prior lines of therapy, race. Make rows, using the same headings for all identified subgroups for TTST. A table is not needed if there are no additional data.”*

**Next sub-prompt:**

*“ now extract the results from these publications and their supplementary materials, ensuring to include all possible subgroups, particularly those documented in figures and tables, from all relevant sections, figures, and tables into one comprehensive table. Complete for complete response (CR), stable disease (SD), partial response (PR) and progressed disease (PD) results.*

*Use the specified headings: Study name (the first author surname and the year the paper was published e.g. Smith 2023, if the clinical trial has a name i.e. CheckMate 123,report this instead), Treatment arm (Extract the drug names for each treatment arm), Treatment line (state first line as 1L, second line as 2L etc), Treatment category (state as platinum chemotherapy, non-platinum chemotherapy, placebo, non-chemotherapy, non-chemotherapy plus chemotherapy), Subgroup name, Analysis set (State the analysis set out of: Intention to treat [ITT], Modified intention to treat [mITT], Per protocol [PP], subgroups. State the term only), Details of analysis set (concisely state details of the analysis set e.g. “All patients randomised to the standard arm with at least one known treatment administration”), Outcome assessor (state how outcomes were assessed in the study out of: "Investigator" or "Independent/central review". State the term only. For example, if the text states "Outcomes were assessed by investigators.", the answer should be "Investigator"), Assessment criteria (State the assessment criteria used to evaluate response. State the term only. For example if the text states "The antitumor response was assessed by Response Evaluation Criteria in Solid Tumors [RECIST] version 1.1.", the answer should be "Response Evaluation Criteria in Solid Tumors (RECIST) version 1.1), Type of timepoint (state the type of timepoint(s) at which outcomes are reported for the treatment arm, for example if a specific date or time is used, like "Month 6" or "01/01/2018" - state “Specific timepoint”. If data is presented after follow-up without a specific timepoint reported, state “Data cut-off/follow-up” only use these two options), Timepoint (State the timepoint(s) at which outcomes are reported for the treatment arm, for example this might look like "Month 6" or a specific date like "01/01/2018" - state only the timepoint. If data is presented after follow-up without a specific timepoint reported, state 'Data cut-off'. For example: If the study reports a median or mean value for an outcome (e.g. median PFS), with data presented after follow-up, the answer should be 'Data cut-off' or a specific data e.g. "01/01/2016" if the date of cut-off is presented. If the study reports a survival outcome at a specific timepoint e.g. 6-month progression free survival (PFS), the answer should be "Month 6". If the study reports a survival outcome at another timepoint e.g. 1-year PFS, report “Year 1” on another row), Date of cut-off (If “Data cut-off/follow-up” is stated in the type of timepoint heading, state the date of the cut-off in this format DD/MM/YYYY. For example if the text states “At data cutoff (January 15, 2020) median follow-up was” report 15/01/2020), Reported? –CR (If CR is reported for this treatment arm and timepoint, state "Y". If not, state "NR"), Definition -CR (State the definition of CR if reported in the publication, as concisely as possible. Just state the definition on its own e.g. "RECIST version 1.1"), Denominator - CR (State the denominator [number at risk] for CR), n, complete response- CR (State the number of patients with CR), %, complete response- CR (State the percentage of patients with CR), p vs comparator- CR (State the p value for this treatment arm and timepoint compared to a comparator for CR), Specify comparator- CR (If a p value for the this treatment arm and timepoint compared to a comparator for CR is reported, report the comparator. Be as concise as possible by stating only the name of the comparator group e.g. "Docetaxel". If this treatment arm and timepoint is not statistically compared to a comparator for CR, or if a p-value is only presented for the hazard ratio (HR), state "NR".), Reported? –SD (If SD is reported for this treatment arm and timepoint, state "Y". If not, state "NR"), Definition -SD (State the definition of SD if reported in the publication, as concisely as possible. Just state the definition on its own e.g. "RECIST version 1.1"), Denominator - SD (State the denominator [number at risk] for SD), n, complete response- SD (State the number of patients with SD), %, complete response- SD (State the percentage of patients with SD), p vs comparator- SD (State the p value for this treatment arm and timepoint compared to a comparator for SD), Specify comparator- SD (If a p value for the this treatment arm and timepoint compared to a comparator for SD is reported, report the comparator. Be as concise as possible by stating only the name of the comparator group e.g. "Docetaxel". If this treatment arm and timepoint is not statistically compared to a comparator for SD, or if a p-value is only presented for the hazard ratio (HR), state "NR"), Reported? –PR (If PR is reported for this treatment arm and timepoint, state "Y". If not, state "NR"), Definition -PR (State the definition of PR if reported in the publication, as concisely as possible. Just state the definition on its own e.g. "RECIST version 1.1"), Denominator - PR (State the denominator [number at risk] for PR), n, complete response- PR (State the number of patients with PR), %, complete response- PR (State the percentage of patients with PR), p vs comparator- PR (State the p value for this treatment arm and timepoint compared to a comparator for PR), Specify comparator- PR (If a p value for the this treatment arm and timepoint compared to a comparator for PR is reported, report the comparator. Be as concise as possible by stating only the name of the comparator group e.g. "Docetaxel". If this treatment arm and timepoint is not statistically compared to a comparator for PR, or if a p-value is only presented for the hazard ratio (HR), state "NR"), Reported? –PD (If PD is reported for this treatment arm and timepoint, state "Y". If not, state "NR"), Definition -PD (State the definition of PD if reported in the publication, as concisely as possible. Just state the definition on its own e.g. "RECIST version 1.1"), Denominator - PD (State the denominator [number at risk] for PD), n, complete response- PD (State the number of patients with PD), %, complete response- PD (State the percentage of patients with PD), p vs comparator- PD (State the p value for this treatment arm and timepoint compared to a comparator for PD), Specify comparator- PD (If a p value for the this treatment arm and timepoint compared to a comparator for PD is reported, report the comparator. Be as concise as possible by stating only the name of the comparator group e.g. "Docetaxel". If this treatment arm and timepoint is not statistically compared to a comparator for PD, or if a p-value is only presented for the HR, state "NR").”*

**Next sub-prompt:**

*“Now make rows, using the same headings for different timepoints for CR, SD, PR and PD. A table is not needed if there are no additional data.”*

**Next sub-prompt:**

*“Now make rows, using the same headings for different assessors for CR, SD, PR and PD. A table is not needed if there are no additional data.”*

**Next sub-prompt:**

*“Now make rows, using the same headings for different analysis sets for CR, SD, PR and PD. A table is not needed if there are no additional data.”*

**Next sub-prompt:**

*“Now identify all possible subgroups reported across the entirety of the publications, including supplementary tables and figures, to ensure comprehensive representation. Examples of potential subgroups include: gender (male or female), age, ECOG performance status, smoking status, severity of disease, tumour stage, mutation status, prior lines of therapy, race. Make rows, using the same headings for all identified subgroups for CR, SD, PR and PD. A table is not needed if there are no additional data.”*

- - 1. Safety Data

**Initial prompt:**

*“You are a systematic reviewer investigating clinical outcomes for endometrial cancer. I am going to provide you with publications for the same study which report clinical outcome data associated with endometrial cancer. I am also going to provide you with supplementary materials if they are available. Extract the results from these publications into a table. Make a new row for each treatment arm identified in the publication as well as each timepoint for which the outcome is reported, and report the corresponding data in the relevant row.*

- *For example, the information and results for treatment arm 1 “Lenvatinib and pembrolizumab " will be reported on row one and the information and results for treatment arm 2 “Placebo” will be reported in the second row. Follow these steps to identify the treatment arms 1. Locate the section or sentence that describes the treatment arms. 2. Identify the different treatments used for each arm. If multiple treatments were used, then separate with a +. For example, if the text states "Patients were randomized 1:1 to receive oral Lenvatinib 500 mg and pembrolizumab 250 mg or oral placebo once per day for a 28-day cycle", the first treatment group should be " Lenvatinib + pembrolizumab " and the second treatment group “placebo”.*
- *Include any data presented for the entire cohort. This could be reported as entire cohort or all patients. If data is reported for all patients in a study, use ‘Entire cohort’ in the treatment arm column. This will be data such as cohort-wide statistics including any of the other categories we will ask you to include. This data is more difficult to pick up as it may not explicitly be described with all patients or entire cohort. You can identify this information by looking for phrases such as ‘a total of [number or %] patients reported [outcome]’, ‘overall [number or %] [outcome] was reported’ or ‘of the [number or %] who [event], [number] were in arm 1 and [number] in arm 2.*
- *Add subgroup data on a new row i.e. if patients receiving the same treatment were further stratified in groups. Examples of subgroups may be histology, biomarker status, administration of the drug (concurrent or sequential).*

*Do you have any questions before I provide you with the paper?”*

**Next sub-prompt:**

*“For the table, use the following column headings: Study name (the first author surname and the year the paper was published e.g. Smith 2023, if the clinical trial has a name i.e. CheckMate 123,report this instead); Treatment arm (Extract the drug names for each treatment arm); Treatment line ( select from 1L, 2L or 1L/2L [if it is a mixed population]).”*

**Next sub-prompt:**

*“ add the following columns: Reported? –all cause mortality (If all-cause mortality is reported for this treatment arm and timepoint, state "Y". If not, state "NR". This could be reported as the number of deaths or people who died); n – all cause mortality (state the number of patients with all-cause mortality); % – all cause mortality (state the percentage of patients with all-cause mortality); Denominator -all cause mortality (state the number of patients who were included in the calculations for all-cause mortality); Reported? –treatment related mortality (If treatment related mortality is reported for this treatment arm and timepoint, state "Y". If not, state "NR"); n – treatment related mortality (state the number of patients with treatment-related mortality); % – treatment related mortality (state the percentage of patients with treatment-related mortality); Denominator -treatment related mortality (state the number of patients who were included in the calculations for treatment related mortality); Reported? –discontinuation overall (If discontinuation is reported for this treatment arm and timepoint, state "Y". If not, state "NR"); n – discontinuation overall (state the number of patients with discontinuation); % – discontinuation overall (state the percentage of patients with discontinuation); Denominator -discontinuation overall (state the number of patients who were included in the calculations for discontinuation); Reported? –discontinuation due to disease progression (If discontinuation due to disease progression is reported for this treatment arm and timepoint, state "Y". If not, state "NR"); n – discontinuation due to disease progression (state the number of patients with discontinuation due to disease progression); % – discontinuation due to disease progression (state the percentage of patients with discontinuation due to disease progression); Denominator -discontinuation due to disease progression (state the number of patients who were included in the calculations for discontinuation due to disease progression); Reported? –discontinuation due to adverse events (If discontinuation due to adverse events is reported for this treatment arm and timepoint, state "Y". If not, state "NR"); n – discontinuation due to adverse events (state the number of patients with discontinuation due to adverse events); % – discontinuation due to adverse events (state the percentage of patients with discontinuation due to adverse events); Denominator -discontinuation due to adverse events (state the number of patients who were included in the calculations for discontinuation due to adverse events); For any information not reported in the publication, respond with "NR". If any numbers have decimals places, include them. Double check that all relevant data have been added to this table from the paper. If anything is missing, add it to the table. IMPORTANT: if percentages are not explicitly reported, do not calculate them.”*

**Next sub-prompt:**

*“Now add; Reported?-overall AEs (If overall AEs is reported for this treatment arm and timepoint, state "Y". If not, state "NR"); n -overall AEs (state the number of patients with overall AEs); % – overall AEs (state the percentage of patients with overall AEs); Denominator -overall AEs (state the number of patients who were included in the calculations for overall AEs); Reported?- grade≥3 AEs (if grade≥3 AEs is reported for this treatment arm and timepoint, state "Y". If not, state "NR"); n – grade 3 AEs (state the number of patients with grade 3 AEs); % – grade 3 AEs (state the percentage of patients with grade 3 AEs); n – grade 4 AEs (state the number of patients with grade 4 AEs); % – grade 4 AEs (state the percentage of patients with grade 4 AEs); n – grade 5 AEs (state the number of patients with grade 5 AEs); % – grade 5 AEs (state the percentage of patients with grade 5 AEs); define category - grade≥3 AEs (if any other category is reported then define here e.g. grade 3–5, grade ≥3); n – grade ≥3 AEs (state the number of patients with grade ≥3 AEs); % – grade ≥3 AEs (state the percentage of patients with grade ≥3 AEs); Denominator -AEs (state the number of patients who were included in the calculations for AEs); Reported?-overall SAEs (If overall SAEs is reported for this treatment arm and timepoint, state "Y". If not, state "NR"); n -overall SAEs (state the number of patients with overall SAEs); % – overall SAEs (state the percentage of patients with overall SAEs); Denominator -overall SAEs (state the number of patients who were included in the calculations for overall SAEs); Reported?-TRAEs (If TRAEs is reported for this treatment arm and timepoint, state "Y". If not, state "NR"); n -TRAEs (state the number of patients with TRAEs); % -TRAEs (state the percentage of patients with TRAEs); Denominator -TRAEs (state the number of patients who were included in the calculations for TRAEs); Reported?- grade≥3 TRAEs (if grade≥3 TRAEs is reported for this treatment arm and timepoint, state "Y". If not, state "NR"); n – grade 3 TRAEs (state the number of patients with grade 3 TRAEs); % – grade 3 TRAEs (state the percentage of patients with grade 3 TRAEs); n – grade 4 TRAEs (state the number of patients with grade 4 TRAEs); % – grade 4 TRAEs (state the percentage of patients with grade 4 TRAEs); n – grade 5 TRAEs (state the number of patients with grade 5 TRAEs); % – grade 5 TRAEs (state the percentage of patients with grade 5 TRAEs); define category - grade≥3 TRAEs (if any other category is reported then define here e.g. grade 3–5, grade ≥3); n – grade ≥3 TRAEs (state the number of patients with grade ≥3 TRAEs); % – grade ≥3 TRAEs (state the percentage of patients with grade ≥3 TRAEs); Denominator -TRAEs (state the number of patients who were included in the calculations for TRAEs); Reported?-TRSAEs (If TRSAEs is reported for this treatment arm and timepoint, state "Y". If not, state "NR"); n -TRSAEs (state the number of patients with TRSAEs); % – TRSAEs (state the percentage of patients with TRSAEs); Denominator -TRSAEs (state the number of patients who were included in the calculations for TRSAEs); Coding dictionary (state the coding dictionary used for AEs e.g. CTCAE). For any information not reported in the publication, respond with "NR". If any numbers have decimals places, include them. Double check that all relevant data have been added to this table from the paper. If anything is missing, add it to the table.”*

**Next sub-prompt:**

*“ add the following columns after treatment line: Subgroup (if all patients are given the same treatment for that row, put ‘All patients’. If different subgroups are reported ,extract into separate rows and specify the subgroup in the subgroup column – this could be biomarker status, age, race, histology, drug administration [concurrent, sequential] etc); Analysis set (State the analysis set out of: Intention to treat [ITT], Modified intention to treat [mITT], Per protocol [PP], subgroups. State the term only); Details of analysis set (concisely state details of the analysis set e.g. “All patients randomised to the standard arm with at least one known treatment administration”); Type of timepoint (State the type of timepoint(s) at which outcomes are reported for the treatment arm, for example if a specific date or time is used, like "Month 6" or "01/01/2018" - state “Specific timepoint”. If data is presented after follow-up without a specific timepoint reported, state “Data cut-off/follow-up” only use these two options); Timepoint (State the timepoint(s) at which outcomes are reported for the treatment arm, for example this might look like "Month 6" or a specific date like "01/01/2018" - state only the timepoint. If data is presented after follow-up without a specific timepoint reported, state 'Data cut-off'. For example: If the study reports a median or mean value for an outcome (e.g. median PFS), with data presented after follow-up, the answer should be 'Data cut-off' or a specific data e.g. "01/01/2016" if the date of cut-off is presented. The timepoint column can also include ‘interim’ and ‘final’ analyses if data are reported at these timepoints. If the study reports a survival outcome at a specific timepoint e.g. 6-month progression free survival (PFS), the answer should be "Month 6". If the study reports a survival outcome at another timepoint e.g. 1-year PFS, report “Year 1” on another row); Date of cut-off (If “Data cut-off/follow-up” is stated in the type of timepoint heading, states the date of the cut-off in this format DD/MM/YYYY); Median follow-up (State the median follow-up time, for the treatment arm. Do not state the units. State only the numerical value e.g. "14.2". If the median follow-up is only reported for the entire cohort, specify this by saying e.g. "Entire cohort: 14.2"); Unit – follow-up (state the units of the median follow-up e.g. months); Median treatment duration or exposure, months (state in months the median treatment duration or exposure for this treatment arm and timepoint).”*

**Next sub-prompt:**

- *“Check for any mistakes. IMPORTANT: if percentages are not explicitly reported, do not calculate them – report as NR.*
- *Cross-check different sections for consistency and completeness of the data extraction.*
- *If treatments contain multiple regimens e.g. "treatment 1 + treatment 2", and data are reported separately for each i.e. discontinuation of treatment 1, and discontinuation of treatment 2.extract this data on separate rows.*
- *Check the supplementary materials and the main pdf. Make sure you only report relevant data, in the correct categories.*
- *Check you have included all relevant time points, including interim analyses. Verify that all relevant data from the study have been captured accurately.*
- *check all data is captured that fits into each of the column heading categories (all-cause mortality; treatment-related mortality; discontinuations overall; discontinuations due to disease progression; discontinuation due to AEs; overall AEs; grade ≥3 AEs; overall serious AEs; treatment-related AEs; grade ≥3 TRAEs; treatment-related serious AEs).”*

**Next sub-prompt:**

*“If treatments contain multiple regimens e.g. "treatment 1 + treatment 2", and data are reported separately for each i.e. discontinuation of treatment 1, and discontinuation of treatment 2.extract this data on separate rows. Specify the treatment arm column as the combination regimen, and use the subgroup column to detail the specific.”*

- - 1. Adverse Event Data

The adverse event prompts were still in development phase when using the endometrial cancer studies testing, and as such, a final set of prompts is not available.

- 1. HCM
     1. Study Characteristics

*“You are a systematic reviewer investigating clinical outcomes for hypertrophic cardiomyopathy (HCM). I am going to provide you with some publications which report clinical outcome data associated with HCM. These publications are all reporting on the same trial. Extract the results from all of these publications into a single table with the following column headings: Study name (the first author surname and the year the paper was published e.g. Smith 2023, if the clinical trial has a name i.e. CheckMate 123report this instead); Study design (out of: RCT, Single-arm trial, Prospective cohort, Retrospective cohort, Case-control study, Cross-sectional study. State the term only); Blinding (If the study is an RCT, state whether it is: Open-label, Single-blind or Double-blind. State the term only); Arms: (State whether the trial was composed of a single arm or multiple arms); Single centre/ multicentre (State whether the study was "single-centre" or "multi-centre". State the term only); Countries (list the countries the study was reported in. List alphabetically and separate countries with a comma e.g.: Belgium, UK, US); Study phase (State the study phase, out of: Phase I, Phase II, Phase III, Phase IV. State one answer); Study funding/sponsor (State the type study sponsor or funder, such as industry, academia, medical groups, or governments, as concisely as possible, will probably be reported at the end of the population under Funding and the specific funding body if specified); Study start date (State the study start date in dd/mm/yyyy format. State one answer); Primary outcome timepoint, date (State the data cut-off for the primary outcome); Primary outcome timepoint, name (State the name, and any key details, for the primary outcome); Primary outcome timepoint, median follow-up (State the median follow-up for the primary outcome); Other timepoints (state any other timepoints that data were reported for. Do not list timepoints if there are no results reported at that timepoint in the article); Inclusion criteria (List the full inclusion criteria. Separate different points with a semi-colon. Do not say "The inclusion criteria were…"); Exclusion criteria (List the full exclusion criteria. Separate different points with a semi-colon. e.g., do not say "The exclusion criteria were…"); N screened (State the number of patients screened for the study. State as one number); N enrolled/randomised (entire cohort) (how many patients were enrolled or randomised in the trial overall); N (patients of relevance to review/in primary analysis set) (How many patients were included in the primary analysis set of the study. This will likely the number of patients assessed for efficacy outcomes [e.g., ITT]. Do not calculate the number if not reported explicitly); Non-obstructive HCM definition (Define in terms of LV wall thickness and peak LVOT pressure); Outcomes assessor (State whether outcomes were: investigator-assessed, independently-assessed or both. State the term only); Treatment 1 (Follow these steps to identify the first treatment arm and extract the drug name: 1. Locate the section or sentence that describes the treatment arms. 2. Identify the first treatment arm described within this section. 3. Extract the name of the drug or drugs used in this first treatment arm along with the dose, frequency (e.g. once daily) and administration route (e.g. oral). 4. If no treatment arm is described or the drug name or names are not reported, respond with "NR". For example: If the text states "Patients were randomized 1:1 to receive oral apatinib 500 mg and gefitinib 250 mg (A + G group) or oral placebo and gefitinib 250 mg (P + G group) once per day for a 28-day cycle", the answer should be "Apatinib and gefitinib". If no information about the first treatment arm is provided, respond with "NR"); Treatment classes (Treatment 1) (select from provided options: Anti-anginal, CMI, ARB, cardiac miotrope, CMI. You can combine these treatment classes if the treatment is a combination of therapies); Treatment 1 (Follow these steps to identify the first treatment arm and extract the drug name: 1. Locate the section or sentence that describes the treatment arms. 2. Identify the first treatment arm described within this section. 3. Extract the name of the drug or drugs used in this first treatment arm along with the dose, frequency (e.g. once daily) and administration route (e.g. oral). 4. If no treatment arm is described or the drug name or names are not reported, respond with "NR". For example: If the text states "Patients were randomized 1:1 to receive oral apatinib 500 mg and gefitinib 250 mg (A + G group) or oral placebo and gefitinib 250 mg (P + G group) once per day for a 28-day cycle", the answer should be "Apatinib and gefitinib". If no information about the first treatment arm is provided, respond with "NR"); Treatment classes (Treatment 2) (select from provided options: Anti-anginal, CMI, ARB, cardiac miotrope, CMI. You can combine these treatment classes if the treatment is a combination of therapies); Control (Follow these steps to identify the first treatment arm and extract the drug name: 1. Locate the section or sentence that describes the treatment arms. 2. Identify the first treatment arm described within this section. 3. Extract the name of the drug or drugs used in this first treatment arm along with the dose, frequency (e.g. QD) and administration route (e.g. oral). 4. If no treatment arm is described or the drug name or names are not reported, respond with "NR". For example: If the text states "Patients were randomized 1:1 to receive oral apatinib 500 mg and gefitinib 250 mg (A + G group) or oral placebo and gefitinib 250 mg (P + G group) once per day for a 28-day cycle", the answer should be "Apatinib and gefitinib". If no information about the first treatment arm is provided, respond with "NR"); Treatment classes (Control) (select from provided options: Anti-anginal, CMI, ARB, cardiac miotrope, CMI, placebo, other. You can combine these treatment classes if the treatment is a combination of therapies). If information is not available, enter NR. Fill out each cell in the table as concisely as possible. Make sure the subject headings are column headings (i.e. a single row for this study).”*

- - 1. Baseline Characteristics

**Initial prompt:**

*“You are a systematic reviewer investigating clinical outcomes for hypertrophic cardiomyopathy (HCM). I am going to provide you with publications reporting clinical outcome data associated with HCM. Extract the results from this publication into a table. Make a new row for each treatment arm identified in the publication, and report the corresponding data in the relevant row. For example, the information and results for treatment arm 1 “Apatinib and gefitinib" will be reported on row one and the information and results for treatment arm 2 “Placebo” will be reported in the second row. Follow these steps to identify the treatment arms 1. Locate the section or sentence that describes the treatment arms. 2. Identify the different treatment arms described within this section with the first treatment arm described first and second treatment arm described second. For example, if the text states "Patients were randomized 1:1 to receive oral apatinib 500 mg and gefitinib 250 mg (A + G group) or oral placebo and gefitinib 250 mg (P + G group) once per day for a 28-day cycle", the first treatment group should be "Apatinib and gefitinib" and the second treatment group “placebo and gefitinib”. For the table use the following headings: Study name (the first author surname and the year the paper was published e.g. Smith 2023, if the clinical trial has a name i.e. CheckMate 123,report this instead); Treatment arm (Extract the drug names for each treatment arm); Treatment arm class (select from provided options: Anti-anginal, CMI, ARB, cardiac miotrope, CMI. You can combine these treatment classes if the treatment is a combination of therapies); Subgroup name (if baseline characteristics are reported for a subgroup of the overall treatment arm/patient population, specify the name of the subgroup here. If the data being extracted is for the overall treatment arm, i.e., not a subgroup, then state "All"); Subgroup characteristic (provide any additional details on the subgroup here. If the data being extracted is for the overall treatment arm, i.e., not a subgroup, then state "NR"); Reported? –age (Is information on age reported for each treatment arm? If yes, state "Y", if no, state "NR"); Mean – age (state the mean age. State as one number without units. E.g., if mean age is 65 years, the answer should be "65". For example: If the text states "Age, mean (range) 55 (40-84)", the answer should be "55" but if the text states "Age, median (range) 55 (40-84)", the answer should be "NR"); Median – age (state the median age. State as one number without units); SD – age (state the standard deviation [SD] for age. State as one number); SE – age (State the standard error [SE] for age. State as one number); IQR Q1 – age (State the lower inter-quartile range [IQR] for age. For example: If the text states "The inter-quartile range [IQR] for age 51–66” the answer should be "51”); IQR Q3– age (State the upper IQR for age. For example: If the text states " inter-quartile range [IQR] for age 51–66” the answer should be "66”).; Range lower limit – age (State the lower range value for age. State as one number. For example: If the text states "Age, median (range), years 60 (27 to 78).", the answer should be "27".); Range upper limit – age (State the upper range value for age. State as one number. For example: If the text states "Age, median (range), years 60 (27 to 78)" the answer should be "78"); Denominator-age (state the number of patients who were included in the calculations for age); p vs comparator – age (State the p value for the treatment groups for age. State as one number." For example, if the text states "Age, median (range), years 60 (27 to 78), p value 0.3", the answer should be "0.3".); Specify comparator- age (If a p value for the treatment arm compared to a comparator for age is reported, report the comparator. Be as concise as possible by stating only the name of the comparator group e.g. "Docetaxel"). If any information is not available, enter NR. Fill out each cell in the table as concisely as possible.”*

**Next sub-prompt:**

*“Now make a table for sex and ethnicity results. As with the table above, make a new row for each treatment arm identified in the publication, and report the corresponding data in the relevant row. For the table use the following headings: Study name (the first author surname and the year the paper was published e.g. Smith 2023, if the clinical trial has a name i.e. CheckMate 123,report this instead); Treatment arm (Extract the drug names for each treatment arm); Treatment arm (Extract the drug names for each treatment arm); Treatment arm class (select from provided options: Anti-anginal, CMI, ARB, cardiac miotrope, CMI. You can combine these treatment classes if the treatment is a combination of therapies); Subgroup name (if baseline characteristics are reported for a subgroup of the overall treatment arm/patient population, specify the name of the subgroup here. If the data being extracted is for the overall treatment arm, i.e., not a subgroup, then state "All"); Subgroup characteristic (provide any additional details on the subgroup here. If the data being extracted is for the overall treatment arm, i.e., not a subgroup, then state "NR"); Reported? – sex (If information on sex (male/female) is reported for the treatment arm, state "Y"); n, males – sex (report the number of males); %, males – sex (report the number of males); n, females – sex (report the number of females); %, females – sex (report the percentage of females); p vs comparator – sex (State the p value for the treatment groups for sex." For example, if the text states "Gender, P 0.480", the answer should be "0.480"); Reported? – race (If information on race or ethnicity is reported for the treatment arm, state "Y"); n, white – race/ethnicity (report the number of white patients); %, white – race/ethnicity (report the percentage of white patients); n, black – race/ethnicity (report the number of black patients); %, black – race/ethnicity (report the percentage of black patients); n, Hispanic – race/ethnicity (report the number of Hispanic patients); %, Hispanic – race/ethnicity (report the percentage of Hispanic patients); n, Asian – race/ethnicity (report the number of Asian patients); %, Asian – race/ethnicity (report the percentage of Asian patients); n, other – race/ethnicity (report the number of other patients); %, other – race/ethnicity (report the percentage of other patients); Denominator-race (state the number of patients who were included in the calculations for race); p vs comparator – race (State the p value for the treatment groups for race. State as one number." For example, if the text states "Race, white, p value 0.3", the answer should be "0.3".); Specify comparator- race (If a p value for the treatment arm compared to a comparator for race is reported, report the comparator. Be as concise as possible by stating only the name of the comparator group e.g. "Docetaxel"). If any information is not available, enter NR. Fill out each cell in the table as concisely as possible.”*

**Next sub-prompt:**

*“Now make a table for BMI and HCM family history results. As with the table above, make a new row for each treatment arm identified in the publication, and report the corresponding data in the relevant row. For the table use the following headings: Study name (the first author surname and the year the paper was published e.g. Smith 2023, if the clinical trial has a name i.e. CheckMate 123,report this instead); Treatment arm (Extract the drug names for each treatment arm); Treatment arm (Extract the drug names for each treatment arm); Treatment arm class (select from provided options: Anti-anginal, CMI, ARB, cardiac miotrope, CMI. You can combine these treatment classes if the treatment is a combination of therapies); Subgroup name (if baseline characteristics are reported for a subgroup of the overall treatment arm/patient population, specify the name of the subgroup here. If the data being extracted is for the overall treatment arm, i.e., not a subgroup, then state "All"); Subgroup characteristic (provide any additional details on the subgroup here. If the data being extracted is for the overall treatment arm, i.e., not a subgroup, then state "NR"); Reported? – BMI (If information on body mass index is reported for the treatment arm, state "Y"); Mean – BMI (state the mean BMI. State as one number without units. E.g., if mean BMI is 65 years, the answer should be "65". For example: If the text states "Age, mean (range) 55 (40-84)", the answer should be "55" but if the text states "Age, median (range) 55 (40-84)", the answer should be "NR"); Median – BMI (state the median BMI. State as one number without units); SD – BMI (state the standard deviation [SD] for BMI. State as one number); SE – BMI (State the standard error [SE] for BMI. State as one number); IQR Q1 – BMI (State the lower inter-quartile range [IQR] for BMI. For example: If the text states "The inter-quartile range [IQR] for BMI 25-30” the answer should be "30”); IQR Q3– age (State the upper IQR for BMI. For example: If the text states " inter-quartile range [IQR] for BMI 25-30” the answer should be "30”).; Range lower limit – BMI (State the lower range value for BMI. State as one number. For example: If the text states "BMI, median (range), years 30 (27 to 30).", the answer should be "27".); Range upper limit – BMI (State the upper range value for BMI. State as one number. For example: If the text states "BMI, median (range), years 30 (27 to 30).", the answer should be "30".); Denominator-age (state the number of patients who were included in the calculations for BMI); p vs comparator – BMI (State the p value for the treatment groups for BMI. State as one number." For example, if the text states "BMI, median (range), (27 to 78), p value 0.3", the answer should be "0.3".); Specify comparator- BMI (If a p value for the treatment arm compared to a comparator for BMI is reported, report the comparator. Be as concise as possible by stating only the name of the comparator group e.g. "Docetaxel"); Reported? – HCM family history (If information on HCM family history is reported for the treatment arm, state "Y"); n – HCM family history (report the number of patients with a family history of HCM); n – HCM family history (report the percentage of patients with a family history of HCM); Denominator-age (state the number of patients who were included in the calculations for HCM family history). If any information is not available, enter NR. Fill out each cell in the table as concisely as possible.”*

**Next sub-prompt:**

*“Now make a table for prior interventions and concurrent/background medications at baseline results. As with the table above, make a new row for each treatment arm identified in the publication, and report the corresponding data in the relevant row. For the table use the following headings: Study name (the first author surname and the year the paper was published e.g. Smith 2023, if the clinical trial has a name i.e. CheckMate 123,report this instead); Treatment arm (Extract the drug names for each treatment arm); Treatment arm (Extract the drug names for each treatment arm); Treatment arm class (select from provided options: Anti-anginal, CMI, ARB, cardiac miotrope, CMI. You can combine these treatment classes if the treatment is a combination of therapies); Subgroup name (if baseline characteristics are reported for a subgroup of the overall treatment arm/patient population, specify the name of the subgroup here. If the data being extracted is for the overall treatment arm, i.e., not a subgroup, then state "All"); Subgroup characteristic (provide any additional details on the subgroup here. If the data being extracted is for the overall treatment arm, i.e., not a subgroup, then state "NR"); Reported? – prior interventions (If information on prior interventions is reported for the treatment arm, state "Y"); n – prior SRT (report the number of patients who were treated with prior SRT); n – prior SRT (report the percentage of patients who were treated with prior SRT); n – prior ICD (report the number of patients who were treated with prior ICD); n – prior ICD (report the percentage of patients who were treated with prior ICD); (List here any other prior interventions received); Denominator-prior intervention (state the number of patients who were included in the calculations for prior intervention); Reported? – medication at baseline (If information on baseline medication is reported for the treatment arm, state "Y"); n – BB (report the number of patients who were treated with baseline beta-blockers); % – BB (report the percentage of patients who were treated with baseline beta-blockers); n – CCB (report the number of patients who were treated with baseline calcium channel blockers); % – CCB (report the percentage of patients who were treated with baseline calcium channel blockers); n – diuretics (report the number of patients who were treated with baseline diuretics); n – diuretics (report the percentage of patients who were treated with baseline diuretics); (List here any other interventions received at baseline); Denominator-medication at baseline (state the number of patients who were included in the calculations for baseline medications). If any information is not available, enter NR. Fill out each cell in the table as concisely as possible.”*

**Next sub-prompt:**

*“Now make a table for HCM genetic testing and NYHA functional class results. As with the table above, make a new row for each treatment arm identified in the publication, and report the corresponding data in the relevant row. For the table use the following headings: Study name (the first author surname and the year the paper was published e.g. Smith 2023, if the clinical trial has a name i.e. CheckMate 123,report this instead); Treatment arm (Extract the drug names for each treatment arm); Treatment arm (Extract the drug names for each treatment arm); Treatment arm class (select from provided options: Anti-anginal, CMI, ARB, cardiac miotrope, CMI. You can combine these treatment classes if the treatment is a combination of therapies); Subgroup name (if baseline characteristics are reported for a subgroup of the overall treatment arm/patient population, specify the name of the subgroup here. If the data being extracted is for the overall treatment arm, i.e., not a subgroup, then state "All"); Subgroup characteristic (provide any additional details on the subgroup here. If the data being extracted is for the overall treatment arm, i.e., not a subgroup, then state "NR"); Reported? – HCM genetic testing (If information on HCM genetic testing is reported for the treatment arm, state "Y"); n – HCM genetic testing (report the number of patients who underwent HCM genetic testing); % – HCM genetic testing (report the percentage of patients who underwent HCM genetic testing); Denominator-genetic testing (state the number of patients who were included in the calculations for HCM genetic testing); Any specific genotypes reported? (state information on the genotypes involved e.g. MYBPC3, MYH7, MYL3, TNNT2, TNNI3, TPM1 or history of known HCM-causing gene mutations); Reported? – NYHA functional class (If information on NYHA functional class is reported for the treatment arm, state "Y"); n – I (NYHA) (report the number of patients who have NYHA functional class I); n – I (NYHA) (report the percentage of patients who have NYHA functional class I); n – II (NYHA) (report the number of patients who have NYHA functional class II); n – II (NYHA) (report the percentage of patients who have NYHA functional class II); n – III (NYHA) (report the number of patients who have NYHA functional class III); n – III (NYHA) (report the percentage of patients who have NYHA functional class III); Denominator-NYHA (state the number of patients who were included in the calculations for NYHA functional class). If any information is not available, enter NR. Fill out each cell in the table as concisely as possible.”*

**Next sub-prompt:**

*“Now make a table for maximum left ventricular wall thickness (WLVWT) and left atrial volume index (LAVI). As with the table above, make a new row for each treatment arm identified in the publication, and report the corresponding data in the relevant row. For the table use the following headings: Study name (the first author surname and the year the paper was published e.g. Smith 2023, if the clinical trial has a name i.e. CheckMate 123,report this instead); Treatment arm (Extract the drug names for each treatment arm); Treatment arm (Extract the drug names for each treatment arm); Treatment arm class (select from provided options: Anti-anginal, CMI, ARB, cardiac miotrope, CMI. You can combine these treatment classes if the treatment is a combination of therapies); Subgroup name (if baseline characteristics are reported for a subgroup of the overall treatment arm/patient population, specify the name of the subgroup here. If the data being extracted is for the overall treatment arm, i.e., not a subgroup, then state "All"); Subgroup characteristic (provide any additional details on the subgroup here. If the data being extracted is for the overall treatment arm, i.e., not a subgroup, then state "NR"); Reported? – MLVWT (If information on maximum left ventricular wall thickness is reported for the treatment arm, state "Y"); Unit (WLVWT) - (state the units of the maximum left ventricular wall thickness e.g. mm); Mean – WLVWT (state the mean WLVWT. State as one number without units. E.g., if mean maximum left ventricular wall thickness is 15 mm, the answer should be "15". For example: If the text states "MLVWT, mean (range) 15 (10-15)", the answer should be "15" but if the text states "MLVWT, median (range) 15 (10-15)", the answer should be "NR"); Median – WLVWT (state the median MLVWT. State as one number without units); SD – MLVWT (state the standard deviation [SD] for MLVWT. State as one number); SE – MLVWT (State the standard error [SE] for WLVWT. State as one number); Denominator-NYHA (state the number of patients who were included in the calculations for MLVWT); Reported? – LAVI (If information on left atrial volume (LAVI) is reported for the treatment arm, state "Y"); Unit (LAVI) - (state the units of the left atrial volume e.g. mL/m2 or mL); Mean – LAVI (state the mean LAVI. State as one number without units. E.g., if mean left atrial volume is 15 mL, the answer should be "15". For example: If the text states "LAVI, mean (range) 15 (10-15)", the answer should be "15" but if the text states "LAVI, median (range) 15 (10-15)", the answer should be "NR"); Median – LAVI (state the median LAVI. State as one number without units); SD – LAVI (state the standard deviation [SD] for LAVI. State as one number); SE – LAVI (State the standard error [SE] for LAVI. State as one number); Denominator-LAVI (state the number of patients who were included in the calculations for LAVI). If any information is not available, enter NR. Fill out each cell in the table as concisely as possible.”*

**Next sub-prompt:**

*“Now make a table for left ventricular (LV) diastolic function and pVO2. As with the table above, make a new row for each treatment arm identified in the publication, and report the corresponding data in the relevant row. For the table use the following headings: Study name (the first author surname and the year the paper was published e.g. Smith 2023, if the clinical trial has a name i.e. CheckMate 123,report this instead); Treatment arm (Extract the drug names for each treatment arm); Treatment arm (Extract the drug names for each treatment arm); Treatment arm class (select from provided options: Anti-anginal, CMI, ARB, cardiac miotrope, CMI. You can combine these treatment classes if the treatment is a combination of therapies); Subgroup name (if baseline characteristics are reported for a subgroup of the overall treatment arm/patient population, specify the name of the subgroup here. If the data being extracted is for the overall treatment arm, i.e., not a subgroup, then state "All"); Subgroup characteristic (provide any additional details on the subgroup here. If the data being extracted is for the overall treatment arm, i.e., not a subgroup, then state "NR"); Reported? – LV diastolic function (If information on maximum LV diastolic function is reported for the treatment arm, state "Y"); Unit (LV diastolic function) - (state the units of the LV diastolic function e.g. mm); Mean – LV diastolic function (state the mean LV diastolic function. State as one number without units. E.g., if mean LV diastolic function is 15 mm, the answer should be "15". For example: If the text states "LV diastolic function, mean (range) 15 (10-15)", the answer should be "15" but if the text states "LV diastolic function, median (range) 15 (10-15)", the answer should be "NR"); Median – LV diastolic function (state the median LV diastolic function. State as one number without units); SD – LV diastolic function (state the standard deviation [SD] for LV diastolic function. State as one number); SE – LV diastolic function (State the standard error [SE] for LV diastolic function. State as one number); Denominator-LV diastolic function (state the number of patients who were included in the calculations for LV diastolic function); Reported? – pVO2 (If information on maximum pVO2 is reported for the treatment arm, state "Y"); Unit (pVO2) - (state the units of the pVO2 e.g. mm); Mean – pVO2 (state the mean pVO2. State as one number without units. E.g., if mean pVO2 is 15 mm, the answer should be "15". For example: If the text states "pVO2, mean (range) 15 (10-15)", the answer should be "15" but if the text states "pVO2, median (range) 15 (10-15)", the answer should be "NR"); Median – pVO2 (state the median LV diastolic function. State as one number without units); SD – pVO2 (state the standard deviation [SD] for pVO2. State as one number); SE – pVO2 (State the standard error [SE] for pVO2. State as one number); Denominator-pVO2 (state the number of patients who were included in the calculations for pVO2). If any information is not available, enter NR. Fill out each cell in the table as concisely as possible.”*

**Next sub-prompt:**

*“Now make a table for Left ventricular ejection fraction (LVEF) and Minute ventilation to carbon dioxide production (VE/VCO2). As with the table above, make a new row for each treatment arm identified in the publication, and report the corresponding data in the relevant row. For the table use the following headings: Study name (the first author surname and the year the paper was published e.g. Smith 2023, if the clinical trial has a name i.e. CheckMate 123,report this instead); Treatment arm (Extract the drug names for each treatment arm); Treatment arm (Extract the drug names for each treatment arm); Treatment arm class (select from provided options: Anti-anginal, CMI, ARB, cardiac miotrope, CMI. You can combine these treatment classes if the treatment is a combination of therapies); Subgroup name (if baseline characteristics are reported for a subgroup of the overall treatment arm/patient population, specify the name of the subgroup here. If the data being extracted is for the overall treatment arm, i.e., not a subgroup, then state "All"); Subgroup characteristic (provide any additional details on the subgroup here. If the data being extracted is for the overall treatment arm, i.e., not a subgroup, then state "NR"); Reported? – LVEF (If information on maximum LVEF is reported for the treatment arm, state "Y"); Unit (LVEF) - (state the units of the LVEF e.g. mm); Mean – LVEF (state the mean LVEF. State as one number without units. E.g., if mean LVEF is 15 mm, the answer should be "15". For example: If the text states "LVEF, mean (range) 15 (10-15)", the answer should be "15" but if the text states "LVEF, median (range) 15 (10-15)", the answer should be "NR"); Median – LVEF (state the median LVEF. State as one number without units); SD – LVEF (state the standard deviation [SD] for LVEF. State as one number); SE – LVEF (State the standard error [SE] for LVEF. State as one number); Denominator-LVEF (state the number of patients who were included in the calculations for LVEF); Reported? – VE/VCO2 (If information on maximum VE/VCO2 is reported for the treatment arm, state "Y"); Unit (VE/VCO2) - (state the units of the VE/VCO2 e.g. mm); Mean – VE/VCO2 (state the mean VE/VCO2. State as one number without units. E.g., if mean VE/VCO2 is 15 mm, the answer should be "15". For example: If the text states "VE/VCO2, mean (range) 15 (10-15)", the answer should be "15" but if the text states "VE/VCO2, median (range) 15 (10-15)", the answer should be "NR"); Median – VE/VCO2 (state the median VE/VCO2. State as one number without units); SD – VE/VCO2 (state the standard deviation [SD] for VE/VCO2. State as one number); SE – VE/VCO2 (State the standard error [SE] for VE/VCO2. State as one number); Denominator-VE/VCO2 (state the number of patients who were included in the calculations for VE/VCO2). If any information is not available, enter NR. Fill out each cell in the table as concisely as possible.”*

**Next sub-prompt:**

*“Now make a table for High-sensitivity cardiac troponin I (hs-cTnI) and N-terminal pro B-type natriuretic peptide (NT-proBNP). As with the table above, make a new row for each treatment arm identified in the publication, and report the corresponding data in the relevant row. For the table use the following headings: Study name (the first author surname and the year the paper was published e.g. Smith 2023, if the clinical trial has a name i.e. CheckMate 123,report this instead); Treatment arm (Extract the drug names for each treatment arm); Treatment arm (Extract the drug names for each treatment arm); Treatment arm class (select from provided options: Anti-anginal, CMI, ARB, cardiac miotrope, CMI. You can combine these treatment classes if the treatment is a combination of therapies); Subgroup name (if baseline characteristics are reported for a subgroup of the overall treatment arm/patient population, specify the name of the subgroup here. If the data being extracted is for the overall treatment arm, i.e., not a subgroup, then state "All"); Subgroup characteristic (provide any additional details on the subgroup here. If the data being extracted is for the overall treatment arm, i.e., not a subgroup, then state "NR"); Reported? – hs-cTnI (If information on maximum hs-cTnI is reported for the treatment arm, state "Y"); Unit (hs-cTnI) - (state the units of the hs-cTnI e.g. mm); Mean – hs-cTnI (state the mean hs-cTnI. State as one number without units. E.g., if mean hs-cTnI is 15 mm, the answer should be "15". For example: If the text states "hs-cTnI, mean (range) 15 (10-15)", the answer should be "15" but if the text states "hs-cTnI, median (range) 15 (10-15)", the answer should be "NR"); Median – hs-cTnI (state the median hs-cTnI. State as one number without units); SD – hs-cTnI (state the standard deviation [SD] for hs-cTnI. State as one number); SE – hs-cTnI (State the standard error [SE] for hs-cTnI. State as one number); Denominator-hs-cTnI (state the number of patients who were included in the calculations for hs-cTnI); Reported? – NT-proBNP levels (If information on maximum NT-proBNP levels is reported for the treatment arm, state "Y"); Unit (NT-proBNP levels) - (state the units of the NT-proBNP levels e.g. mm); Mean – NT-proBNP levels (state the mean NT-proBNP levels. State as one number without units. E.g., if mean NT-proBNP levels is 15 mm, the answer should be "15". For example: If the text states "NT-proBNP levels, mean (range) 15 (10-15)", the answer should be "15" but if the text states "NT-proBNP levels, median (range) 15 (10-15)", the answer should be "NR"); Median – NT-proBNP levels (state the median NT-proBNP levels. State as one number without units); SD – NT-proBNP levels (state the standard deviation [SD] for NT-proBNP levels. State as one number); SE – NT-proBNP levels (State the standard error [SE] for NT-proBNP levels. State as one number); Denominator-NT-proBNP levels (state the number of patients who were included in the calculations for NT-proBNP levels). If any information is not available, enter NR. Fill out each cell in the table as concisely as possible.”*

**Next sub-prompt:**

*“Now make a table for comorbidities at baseline results. As with the table above, make a new row for each treatment arm identified in the publication, and report the corresponding data in the relevant row. For the table use the following headings: Study name (the first author surname and the year the paper was published e.g. Smith 2023, if the clinical trial has a name i.e. CheckMate 123,report this instead); Treatment arm (Extract the drug names for each treatment arm); Treatment arm (Extract the drug names for each treatment arm); Treatment arm class (select from provided options: Anti-anginal, CMI, ARB, cardiac miotrope, CMI. You can combine these treatment classes if the treatment is a combination of therapies); Subgroup name (if baseline characteristics are reported for a subgroup of the overall treatment arm/patient population, specify the name of the subgroup here. If the data being extracted is for the overall treatment arm, i.e., not a subgroup, then state "All"); Subgroup characteristic (provide any additional details on the subgroup here. If the data being extracted is for the overall treatment arm, i.e., not a subgroup, then state "NR"); Reported? – comorbidities (If information on baseline comorbidities is reported for the treatment arm, state "Y"); n – Anaemia (report the number of patients who had baseline anaemia); % – Anaemia (report the percentage of patients who had baseline anaemia); n – AF (report the number of patients who had baseline Atrial fibrillation [AF]); % – AF (report the percentage of patients who had baseline Atrial fibrillation [AF]); n – COPD (report the number of patients who had baseline Chronic obstructive pulmonary disease [COPD]); % – COPD (report the percentage of patients who had baseline Chronic obstructive pulmonary disease [COPD]); Denominator-comorbidities (state the number of patients who were included in the calculations for comorbidities). If any information is not available, enter NR. Fill out each cell in the table as concisely as possible.”*

- - 1. Efficacy

**Initial prompt:**

*“You are a systematic reviewer investigating clinical outcomes for hypertrophic cardiomyopathy (HCM). I will provide you with publications and their supplementary materials that report clinical outcome data associated with Acetract the results from these publications and their supplementary materials, ensuring to include all possible subgroups, particularly those documented in figures and tables, from all relevant sections, figures, and tables into one comprehensive table. Separate the following onto new rows: different treatment arms. Follow these steps:*

1. *Identify Treatment Arms:*

*Locate the section or sentence that describes the treatment arms.*

*Identify the different treatment arms described within this section with the first treatment arm described first and the second treatment arm described second. For example, if the text states "Patients were randomized 1:1 to receive oral apatinib 500 mg and gefitinib 250 mg (A + G group) or oral placebo and gefitinib 250 mg (P + G group) once per day for a 28-day cycle", the first treatment group should be "Apatinib and gefitinib" and the second treatment group “Placebo and gefitinib”.*

1. *Table Formatting:*

*Use the specified headings: Study name (the first author surname and the year the paper was published e.g. Smith 2023, if the clinical trial has a name i.e. CheckMate 123,report this instead), Treatment arm (Extract the drug names for each treatment arm), Treatment class (Placebo, CMI, ARB, Cardiac miotrope, Anti-anginal, etc), Subgroup name, Subgroup description, Analysis set (State the analysis set out of: Intention to treat [ITT], Modified intention to treat [mITT], Per protocol [PP], subgroups. State the term only), Details of analysis set (concisely state details of the analysis set e.g. “All patients randomised to the standard arm with at least one known treatment administration”), Outcome assessor (state how outcomes were assessed in the study out of: "Investigator" or "Independent/central review". State the term only. For example, if the text states "Outcomes were assessed by investigators.", the answer should be "Investigator"), Type of timepoint (state the type of timepoint(s) at which outcomes are reported for the treatment arm, for example if a specific date or time is used, like "Month 6" or "01/01/2018" - state “Specific timepoint”. If data is presented after follow-up without a specific timepoint reported, state “Data cut-off/follow-up” only use these two options), Timepoint (State the timepoint(s) at which outcomes are reported for the treatment arm, for example this might look like "Month 6" or a specific date like "01/01/2018" - state only the timepoint. If data is presented after follow-up without a specific timepoint reported, state 'Data cut-off'. For example: If the study reports a median or mean value for an outcome (e.g. median Left ventricular ejection fraction (LVEF)), with data presented after follow-up, the answer should be 'Data cut-off' or a specific data e.g. "01/01/2016" if the date of cut-off is presented. If the study reports an outcome at a specific timepoint e.g. 6-month LVEF, the answer should be "Month 6". If the study reports an outcome at another timepoint e.g. 1-year LVEF, report “Year 1” on another row), Date of cut-off (If “Data cut-off/follow-up” is stated in the type of timepoint heading, state the date of the cut-off in this format DD/MM/YYYY. For example if the text states “At data cutoff (January 15, 2020) median follow-up was” report 15/01/2020), Median follow-up (State the median follow-up time, for the treatment arm), Reported? –LVEF (If LVEF is reported for this row, state "Y"), Definition -LVEF, Denominator -LVEF, Units- LVEF (State the unit used for LVEF, e.g. % or ml/kg/min etc. State the unit only), Median -LVEF, Mean -LVEF, SE- LVEF, SD -LVEF, IQR, Q1-LVEF, IQR, Q3 -LVEF, 95% CI, lower -LVEF, 95% CI, upper -LVEF, p vs baseline -LVEF, p vs comparator-LVEF, Specify comparator- LVEF (If a p value for the treatment arm compared to a comparator for LVEF is reported, report the comparator. Be as concise as possible by stating only the name of the comparator group e.g. "Docetaxel". If this treatment arm and timepoint is not statistically compared to a comparator for LVEF state "NR"), mean change from baseline in LVEF, SD change from baseline in LVEF, SE change from baseline in LVEF, 95% CI, lower change from baseline in LVEF, 95% CI, upper change from baseline in LVEF, median change from baseline in LVEF, IQR lower change from baseline in LVEF, IQR upper change from baseline in LVEF, p vs baseline, p vs comparator change from baseline in LVEF, Specify comparator change from baseline in LVEF*

1. *Ensure Completeness and Specificity:*

*Double-check that all LVEF data and subgroup analyses from the entire documents are thoroughly represented in the table, with separate rows for each treatment arm as needed.*

*If any information is not reported, respond with "NR". Be meticulous in including all data in the figures and tables in the table to ensure comprehensiveness.”*

**Next sub-prompt:**

*“Now make table rows, using the same headings and format for different timepoints. A table is not needed if there are no additional data.”*

**Next sub-prompt:**

*“Now make table rows, using the same headings and format for different assessors so that “Investigator" or "Independent/central review" are extracted if needed. A table is not needed if there are no additional data.”*

**Next sub-prompt:**

*“Now make table rows, using the same headings for different analysis sets. A table is not needed if there are no additional data.”*

**Next sub-prompt:**

*“Now identify all possible subgroups reported across the entirety of the publications, including supplementary tables and figures, to ensure comprehensive representation. Examples of potential subgroups include: gender (male or female), age, smoking status, severity of disease, mutation status, prior lines of therapy, race. Make rows, using the same headings for all identified subgroups. A table is not needed if there is no additional data.”*

**Next sub-prompt:**

*“Now make the same original table, but for LV diastolic function (E/e') instead of LVEF.”*

**Next sub-prompt:**

*“Now make rows, using the same headings for different timepoints for LV diastolic function (E/e'). A table is not needed if there are no additional data.”*

**Next sub-prompt:**

*“Now make rows, using the same headings for different assessors for LV diastolic function (E/e'). A table is not needed if there are no additional data.”*

**Next sub-prompt:**

*“Now make rows, using the same headings for different analysis sets for LV diastolic function (E/e'). A table is not needed if there are no additional data.”*

**Next sub-prompt:**

*“Now identify all possible subgroups reported across the entirety of the publications, including supplementary tables and figures, to ensure comprehensive representation. Examples of potential subgroups include: gender (male or female), age, smoking status, severity of disease, mutation status. Make rows, using the same headings for all identified subgroups for LV diastolic function (E/e'). A table is not needed if there are no additional data.”*

**Next sub-prompt:**

*“Now make the same original table, but for peak oxygen consumption (PVO2) instead of LVEF.”*

**Next sub-prompt:**

*“Now make rows, using the same headings for different timepoints for PVO2. A table is not needed if there are no additional data.”*

**Next sub-prompt:**

*“Now make rows, using the same headings for different assessors for PVO2. A table is not needed if there are no additional data.”*

**Next sub-prompt:**

*“Now make rows, using the same headings for different analysis sets for PVO2. A table is not needed if there are no additional data.”*

**Next sub-prompt:**

*“Now identify all possible subgroups reported across the entirety of the publications, including supplementary tables and figures, to ensure comprehensive representation. Examples of potential subgroups include: gender (male or female), age, smoking status, severity of disease, mutation status, prior lines of therapy, race. Make rows, using the same headings for all identified subgroups for PVO2. A table is not needed if there are no additional data.”*

**Next sub-prompt:**

*“Now make the same original table, but for N-terminal pro B-type natriuretic peptide levels (NT-proBNP levels) instead of LVEF.”*

**Next sub-prompt:**

*“Now make rows, using the same headings for different timepoints for NT-proBNP levels. A table is not needed if there are no additional data.”*

**Next sub-prompt:**

*“Now make rows, using the same headings for different assessors for NT-proBNP levels. A table is not needed if there are no additional data.”*

**Next sub-prompt:**

*“Now make rows, using the same headings for different analysis sets for NT-proBNP levels. A table is not needed if there are no additional data.”*

**Next sub-prompt:**

*“Now identify all possible subgroups reported across the entirety of the publications, including supplementary tables and figures, to ensure comprehensive representation. Examples of potential subgroups include: gender (male or female), age, smoking status, severity of disease, mutation status, prior lines of therapy, race. Make rows, using the same headings for all identified subgroups for NT-proBNP levels. A table is not needed if there are no additional data.”*

**Next sub-prompt:**

*“ now extract New York Heart Association (NYHA) functional class results from the publications and their supplementary materials, ensuring to include all possible subgroups, particularly those documented in figures and tables, from all relevant sections, figures, and tables into one comprehensive table.*

*Use the specified headings: Study name (the first author surname and the year the paper was published e.g. Smith 2023, if the clinical trial has a name i.e. CheckMate 123,report this instead), Treatment arm (Extract the drug names for each treatment arm), Treatment class (Placebo, CMI, ARB, Cardiac miotrope, Anti-anginal, etc), Subgroup name, Subgroup description, Analysis set (State the analysis set out of: Intention to treat [ITT], Modified intention to treat [mITT], Per protocol [PP], subgroups. State the term only), Details of analysis set (concisely state details of the analysis set e.g. “All patients randomised to the standard arm with at least one known treatment administration”), Outcome assessor (state how outcomes were assessed in the study out of: "Investigator" or "Independent/central review". State the term only. For example, if the text states "Outcomes were assessed by investigators.", the answer should be "Investigator"), Type of timepoint (state the type of timepoint(s) at which outcomes are reported for the treatment arm, for example if a specific date or time is used, like "Month 6" or "01/01/2018" - state “Specific timepoint”. If data is presented after follow-up without a specific timepoint reported, state “Data cut-off/follow-up” only use these two options), Timepoint (State the timepoint(s) at which outcomes are reported for the treatment arm, for example this might look like "Month 6" or a specific date like "01/01/2018" - state only the timepoint. If data is presented after follow-up without a specific timepoint reported, state 'Data cut-off'. For example: If the study reports a median or mean value for an outcome (e.g. median LVEF), with data presented after follow-up, the answer should be 'Data cut-off' or a specific data e.g. "01/01/2016" if the date of cut-off is presented. If the study reports an outcome at a specific timepoint e.g. 6-month LVEF, the answer should be "Month 6". If the study reports an outcome at another timepoint e.g. 1-year LVEF, report “Year 1” on another row), Date of cut-off (If “Data cut-off/follow-up” is stated in the type of timepoint heading, state the date of the cut-off in this format DD/MM/YYYY. For example if the text states “At data cutoff (January 15, 2020) median follow-up was” report 15/01/2020), Median follow-up (State the median follow-up time, for the treatment arm), Reported? –NYHA (If NYHA is reported for this treatment arm and timepoint, state "Y". If not, state "NR"), Denominator - NYHA (State the denominator for NYHA), n, NYHA I (State the number of patients in NYHA class I), %, NYHA I (State the percentage of patients in NYHA class I), n, NYHA II (State the number of patients in NYHA class II), %, NYHA III (State the percentage of patients in NYHA class III), n, NYHA IV (State the number of patients in NYHA class IV), %, NYHA IV (State the percentage of patients in NYHA IV), n, ≥1 NYHA class improvement (State the number of patients with ≥1 NYHA class improvement), %, ≥1 NYHA class improvement (State the percentage of patients with ≥1 NYHA class improvement), p vs baseline– NYHA, p vs comparator- NYHA (State the p value for this treatment arm and timepoint compared to a comparator for NYHA), Specify comparator- NYHA (If a p value for the this treatment arm and timepoint compared to a comparator for NYHA is reported, report the comparator. Be as concise as possible by stating only the name of the comparator group e.g. "Docetaxel". If this treatment arm and timepoint is not statistically compared to a comparator for NYHA).”*

**Next sub-prompt:**

*“Now make rows, using the same headings for different timepoints for NYHA class. A table is not needed if there are no additional data.”*

**Next sub-prompt:**

*“Now make rows, using the same headings for different assessors for NYHA class. A table is not needed if there are no additional data.”*

**Next sub-prompt:**

*“Now make rows, using the same headings for different analysis sets for NYHA class. A table is not needed if there are no additional data.”*

**Next sub-prompt:**

*“Now identify all possible subgroups reported across the entirety of the publications, including supplementary tables and figures, to ensure comprehensive representation. Examples of potential subgroups include: gender (male or female), age, smoking status, severity of disease, mutation status, prior lines of therapy, race. Make rows, using the same headings for all identified subgroups for NYHA class. A table is not needed if there are no additional data.”*

- - 1. Safety

**Initial prompt:**

*“You are a systematic reviewer investigating safety outcomes for hypertrophic cardiomyopathy (HCM). I have provided you with publications for the same study which report safety outcome data associated with HCM. I have also provided you with supplementary materials if they are available. Extract the results from these publications into a table. Make a new row for each treatment arm identified in the publication as well as each timepoint for which the outcome is reported, and report the corresponding data in the relevant row.*

- *For example, the information and results for treatment arm 1 “Mavacamten" will be reported on row one and the information and results for treatment arm 2 “Placebo” will be reported in the second row. Follow these steps to identify the treatment arms 1. Locate the section or sentence that describes the treatment arms. 2. Identify the different treatments used for each arm. If multiple treatments were used, then separate with a +. For example, if the text states "Patients were randomized 1:1 to receive oral Lenvatinib 500 mg and pembrolizumab 250 mg or oral placebo once per day for a 28-day cycle", the first treatment group should be " Lenvatinib + pembrolizumab " and the second treatment group “placebo”.*
- *Include any data presented for the entire cohort. This could be reported as entire cohort or all patients. If data is reported for all patients in a study, use ‘Entire cohort’ in the treatment arm column. This will be data such as cohort-wide statistics including any of the other categories we will ask you to include. This data is more difficult to pick up as it may not explicitly be described with all patients or entire cohort. You can identify this information by looking for phrases such as ‘a total of [number or %] patients reported [outcome]’, ‘overall [number or %] [outcome] was reported’ or ‘of the [number or %] who [event], [number] were in arm 1 and [number] in arm 2.*
- *Add subgroup data on a new row i.e. if patients receiving the same treatment were further stratified in groups. Examples of subgroups may be histology, biomarker status, administration of the drug (concurrent or sequential).*

*For the table, use the following column headings: Study name (the first author surname and the year the paper was published e.g. Smith 2023, if the clinical trial has a name i.e. CheckMate 123,report this instead); Treatment arm (Extract the drug names for each treatment arm); Treatment arm class (e.g. placebo, CMI, ARB). Reported? –all cause mortality (If all-cause mortality is reported for this treatment arm and timepoint, state "Y". If not, state "NR". This could be reported as the number of deaths or people who died); n – all cause mortality (state the number of patients with all-cause mortality); % – all cause mortality (state the percentage of patients with all-cause mortality); Denominator -all cause mortality (state the number of patients who were included in the calculations for all-cause mortality); Reported? –cardiovascular (CV) related mortality (If treatment related mortality is reported for this treatment arm and timepoint, state "Y". If not, state "NR"); n – treatment related mortality (state the number of patients with treatment-related mortality); % – treatment related mortality (state the percentage of patients with treatment-related mortality); Denominator -treatment related mortality (state the number of patients who were included in the calculations for treatment related mortality); Reported? –all cause hospitalisations (If all-cause hospitalisations is reported for this treatment arm and timepoint, state "Y". If not, state "NR"); definition – all cause hospitalisations (state the definition of all-cause hospitalisations); n – all cause hospitalisations (state the number of patients with all-cause hospitalisations); % – all cause hospitalisations (state the percentage of patients with all-cause hospitalisations); Denominator -all cause hospitalisations (state the number of patients who were included in the calculations for all-cause hospitalisations); Reported? –CV-related hospitalisations (If CV-related hospitalisations is reported for this treatment arm and timepoint, state "Y". If not, state "NR"); definition – CV-related hospitalisations (state the definition of CV-related hospitalisations); n – CV-related hospitalisations (state the number of patients with CV-related hospitalisations); % – CV-related hospitalisations (state the percentage of patients with CV-related hospitalisations); Denominator - CV-related hospitalisations (state the number of patients who were included in the calculations for CV-related hospitalisations); Reported? –Implantable cardiac device (ICD) use or discharge (If ICD use or discharge is reported for this treatment arm and timepoint, state "Y". If not, state "NR"); definition – ICD use or discharge (state the definition of ICD use or discharge); n – ICD use or discharge (state the number of patients with ICD use or discharge); % – ICD use or discharge (state the percentage of patients with ICD use or discharge); Denominator - ICD use or discharge (state the number of patients who were included in the calculations for ICD use or discharge); Reported? –discontinuation overall (If discontinuation is reported for this treatment arm and timepoint, state "Y". If not, state "NR"); n – discontinuation overall (state the number of patients with discontinuation); % – discontinuation overall (state the percentage of patients with discontinuation); Denominator -discontinuation overall (state the number of patients who were included in the calculations for discontinuation); Reported? –discontinuation due to adverse events (If discontinuation due to adverse events is reported for this treatment arm and timepoint, state "Y". If not, state "NR"); n – discontinuation due to adverse events (state the number of patients with discontinuation due to adverse events); % – discontinuation due to adverse events (state the percentage of patients with discontinuation due to adverse events); Denominator -discontinuation due to adverse events (state the number of patients who were included in the calculations for discontinuation due to adverse events); For any information not reported in the publication, respond with "NR". If any numbers have decimals places, include them. Double check that all relevant data have been added to this table from the paper. If anything is missing, add it to the table. IMPORTANT: if percentages are not explicitly reported, do not calculate them.”*

**Next sub-prompt:**

*“Now add; Reported? - overall AEs (If overall AEs is reported for this treatment arm and timepoint, state "Y". If not, state "NR"); n -overall AEs (state the number of patients with overall AEs); % – overall AEs (state the percentage of patients with overall AEs); Denominator -overall AEs (state the number of patients who were included in the calculations for overall AEs); Reported?-overall SAEs (If overall SAEs is reported for this treatment arm and timepoint, state "Y". If not, state "NR"); n -overall SAEs (state the number of patients with overall SAEs); % – overall SAEs (state the percentage of patients with overall SAEs); Denominator -overall SAEs (state the number of patients who were included in the calculations for overall SAEs); Reported?-Treatment emergent adverse events (TEAEs) (If TEAEs is reported for this treatment arm and timepoint, state "Y". If not, state "NR"); grade-TEAEs (state the grade of AE); n -TEAEs (state the number of patients with TEAEs); % -TEAEs (state the percentage of patients with TEAEs); Denominator -TEAEs (state the number of patients who were included in the calculations for TEAEs); Reported?-Treatment emergent serious adverse events (TESAEs) (If TESAEs is reported for this treatment arm and timepoint, state "Y". If not, state "NR"); n -TESAEs (state the number of patients with TESAEs); % -TESAEs (state the percentage of patients with TESAEs); Denominator -TESAEs (state the number of patients who were included in the calculations for TESAEs); Reported?-Treatment related serious adverse event (TRSAEs) (If TRSAEs is reported for this treatment arm and timepoint, state "Y". If not, state "NR"); n -TRSAEs (state the number of patients with TRSAEs); % – TRSAEs (state the percentage of patients with TRSAEs); Denominator -TRSAEs (state the number of patients who were included in the calculations for TRSAEs). For any information not reported in the publication, respond with "NR". If any numbers have decimals places, include them. Double check that all relevant data have been added to this table from the paper. If anything is missing, add it to the table.”*

**Next sub-prompt:**

*“ add the following columns after treatment line: Subgroup (if all patients are given the same treatment for that row, put ‘All patients’. If different subgroups are reported, extract into separate rows and specify the subgroup in the subgroup column – this could be biomarker status, age, race, histology, drug administration [concurrent, sequential] etc); Analysis set (State the analysis set out of: Intention to treat [ITT], Modified intention to treat [mITT], Per protocol [PP], subgroups. State the term only); Details of analysis set (concisely state details of the analysis set e.g. “All patients randomised to the standard arm with at least one known treatment administration”); Type of timepoint (State the type of timepoint(s) at which outcomes are reported for the treatment arm, for example if a specific date or time is used, like "Month 6" or "01/01/2018" - state “Specific timepoint”. If data is presented after follow-up without a specific timepoint reported, state “Data cut-off/follow-up” only use these two options); Timepoint (State the timepoint(s) at which outcomes are reported for the treatment arm, for example this might look like "Month 6" or a specific date like "01/01/2018" - state only the timepoint. If data is presented after follow-up without a specific timepoint reported, state 'Data cut-off'. For example: If the study reports a median or mean value for an outcome (e.g. median PFS), with data presented after follow-up, the answer should be 'Data cut-off' or a specific data e.g. "01/01/2016" if the date of cut-off is presented. The timepoint column can also include ‘interim’ and ‘final’ analyses if data are reported at these timepoints. If the study reports a survival outcome at a specific timepoint e.g. 6-month progression free survival (PFS), the answer should be "Month 6". If the study reports a survival outcome at another timepoint e.g. 1-year PFS, report “Year 1” on another row); Date of cut-off (If “Data cut-off/follow-up” is stated in the type of timepoint heading, states the date of the cut-off in this format DD/MM/YYYY); Median follow-up (State the median follow-up time, for the treatment arm. Do not state the units. State only the numerical value e.g. "14.2". If the median follow-up is only reported for the entire cohort, specify this by saying e.g. "Entire cohort: 14.2"); Unit – follow-up (state the units of the median follow-up e.g. months); Median treatment duration or exposure, months (state in months the median treatment duration or exposure for this treatment arm and timepoint).”*

**Next sub-prompt:**

- *“Check for any mistakes. IMPORTANT: if percentages are not explicitly reported, do not calculate them – report as NR.*
- *Cross-check different sections for consistency and completeness of the data extraction.*
- *If treatments contain multiple regimens e.g. "treatment 1 + treatment 2", and data are reported separately for each i.e. discontinuation of treatment 1, and discontinuation of treatment 2.extract this data on separate rows.*
- *Check the supplementary materials and the main pdf. Make sure you only report relevant data, in the correct categories.*
- *Check you have included all relevant time points, including interim analyses. Verify that all relevant data from the study have been captured accurately.*
- *check all data is captured that fits into each of the column heading categories (all-cause mortality; CV-related mortality; all cause hospitalisations; CV-related hospitalisations; ICD use or discharge; discontinuations overall; discontinuation due to AEs; overall AEs; overall serious AEs; Treatment emergent adverse events; Treatment emergent serious adverse events; treatment-related serious AEs).”*

**Next sub-prompt:**

*“If treatments contain multiple regimens e.g. "treatment 1 + treatment 2", and data are reported separately for each i.e. discontinuation of treatment 1, and discontinuation of treatment 2.extract this data on separate rows. Specify the treatment arm column as the combination regimen, and use the subgroup column to detail the specific.”*

- - 1. Adverse Events

**Initial prompt:**

*“You are a systematic reviewer investigating clinical outcomes for hypertrophic cardiomyopathy (HCM). I am going to provide you with publications which report clinical outcome data associated with HCM. I am also going to provide you with supplementary materials. Extract the results from this publication into a table. Make a new row for each treatment arm identified in the publication and each timepoint for which the outcome is reported -reporting the corresponding data in the relevant row. For example, the information and results for treatment arm 1 “Apatinib and gefitinib" will be reported on row one and the information and results for treatment arm 2 “Placebo” will be reported in the second row. Follow these steps to identify the treatment arms 1. Locate the section or sentence that describes the treatment arms. 2. Identify the different treatment arms described within this section with the first treatment arm described first and second treatment arm described second. For example, if the text states "Patients were randomized 1:1 to receive oral apatinib 500 mg and gefitinib 250 mg (A + G group) or oral placebo and gefitinib 250 mg (P + G group) once per day for a 28-day cycle", the first treatment group should be "Apatinib and gefitinib" and the second treatment group “placebo and gefitinib”. If any numbers have decimals places, include them. Use "NR" for any unreported data/empty cells; there should be no missing or empty columns. Do not perform any calculations. Add separate subgroup data on a new row i.e. if patients receiving the same treatment were further stratified in groups; examples of subgroups may be systolic/diastolic blood pressure, functional or PRO classification at baseline, drug concentration in plasma. For the table, use the following column headings: Study name (the first author surname and the year the paper was published e.g. Smith 2023, if the clinical trial has a name i.e. CheckMate 123,report this instead); Treatment arm (Extract the drug names for each treatment arm) ; Treatment arm description (Extract additional details for the treatment, including dose and frequency, e.g., Mavacamten, oral, once daily, initial dose 5 mg, titrated at Week 6 to target plasma levels of 200 or 500 ng/ml (using dose strengths of 2.5, 5, 10, or 15 mg); Treatment arm class (Specify the treatment class for the drug. Choose from: Cardiac mitotrope, CCB, ARB, Selective cardiac late sodium current inhibitor, anti-anginal, diuretic agent, BB, heart failure medication, heart transplantation, CMI + BB/CCB, BB + CCB, ARB + BB/CCB, Class IA anti-arrhythmic, Class IA anti-arrhythmic/CCB, Placebo, Surgical procedure, Other, CMI or placebo); Cohort/Subgroup name (state ‘All’ if the total cohort is reported, or specify the subgroup name. If data on non-obstructive HCM is reported as a subgroup of the overall cohort data, specify "non-obstructive HCM"); Cohort/Subgroup description (Provide any additional details on the subgroup, if available); N in the analysis set (Specify the number of patients evaluated for analysis for this treatment arm [and subgroup]); Analysis set (choose from all patients, ITT, per-protocol, modified ITT, safety population, or NR); Detail of analysis set; Type of timepoint (choose from data cut-off/follow-up or specific timepoint); Timepoint (specify the timepoint which the data were collected at); Date of data cut-off (only applicable if the type of timepoint was ‘data cut-off/follow-up. Use the format DD/MM/YYYY); Type of follow-up measure (E.g., mean or median); Actual follow-up; Uncertainty for actual follow-up (e.g., range, standard deviation); Median treatment duration or exposure, months; Coding dictionary used for AE classifications (this will likely be reported in the Methods section of the publication. For example, this could be “Common Terminology Criteria for Adverse Events”); AE Grade ( first check for a table reporting any AEs which is likely to report the AE grades. Include all AE grades reported: these could be individual grades or categorised e.g. any or all grades, grade X, GX, Grade X-X, Grade X/X, Grade ≥X [where X is any number between 0 and 5], serious, moderate, any grade. These are only a few examples so present this data how the publication reports it).”*

**Next sub-prompt:**

*“ now generate the same table, but add subsequent column headings for the following AEs: Stroke; Transient ischaemic attack (TIA); Acute myocardial infarction (MI); Atrioventricular (AV) nodal block; Left bundle branch block; Ventricular septal defect; Aortic regurgitation; Acute renal failure; Cardiac/heart failure ; Atrial Fibrillation (incident); Arrythmias; Major Bleeds; Ventricular tachyarrhythmias; Syncope. The column should be named as the specific AE (e.g., arrythmias) and ‘Reported?’ (If the specific AE is reported for this treatment arm and timepoint, state "Y". If not, state "NR"). If multiple AE grades are reported for the same adverse event, separate this onto a new row. Each row should only include one AE grade. For example, if alopecia is reported as grade 1, grade 2 and grade 3. This should be on three rows. If anaemia is also reported as grade 2, this can be on the same row as the alopecia grade 2. If any of these AEs are listed in the publication, but there were 0 cases, report the AE as reported (“Y”).”*

**Next sub-prompt:**

*“For each of the specific AEs, after the ‘Reported?’ column add the following columns: n (for number of patients with the AE), % (percentage of patients with the AE), Denominator (of treatment arm). Each AE should have four columns: Reported, n, %, denominator. Followed by four columns for the next AE etc. double check you have included data for all grades in the table. Data may be presented in the text, as well as in tables. If the ‘Reported?’ column is NR, and there are no data on n and %,add NR in the denominator column. If any of these AEs are listed in the publication, but there were 0 cases, report the AE as reported (“Y”), and use ‘0’ for number of patients and percentage of patients. Give this table subheading ‘Table 1’.”*

**Next sub-prompt:**

*“ now generate the same table, but for the following AEs: Seizures; Dyspnoea; Headache; Dizziness; Alopecia; Diarrhoea; Fatigue; Parosmia; Palpitations; Nausea; Acute left ventricular failure; Ventricular dysfunction; Ejection fraction decreased; Any other major adverse cardiovascular event (MACE).Give this table subheading 'Table 2'.”*

**Next sub-prompt:**

*“Now check the publication for any other grades of the specific AEs (e.g. Grade 4, Grade 5, Grade 1-2, Grade 1/2, Grade 3/4, Grade 3-4, Grade ≥3) reported in the publication that are not currently captured in Table 1 or Table 2 . Add the data for these specific AEs into each table as new rows, if reported.”*

**Next sub-prompt:**

*" now review each table above with the following points, and provide a final, corrected version of each. Add ‘[Final]’ to the subheading of each table.*

- *The table should be complete and comprehensive, all AEs should be presented.*
- *Within each table, the number of columns should be consistent across each row, with four columns for each AE, ensuring all columns are filled for every row. Use ""NR"" (Not Reported) consistently for missing data to maintain table structure.*
- *Within each table, now manually count the number of columns in each row, and then correct so that there are an equal number of columns in each row to maintain table structure*
- *In the markdown, ensure that each row has the exact number of '|' characters to match the number of columns in the header, and that there are no extraneous spaces or line breaks within cells."*
